# Single cell transcriptomic analysis of human amnion identifies cell-specific signatures associated with membrane rupture and parturition

**DOI:** 10.1186/s13578-022-00797-4

**Published:** 2022-05-18

**Authors:** Wang-Sheng Wang, Yi-Kai Lin, Fan Zhang, Wen-Jia Lei, Fang Pan, Ya-Nan Zhu, Jiang-Wen Lu, Chu-Yue Zhang, Qiong Zhou, Hao Ying, Kang Sun

**Affiliations:** 1 Center for Reproductive Medicine, Ren Ji Hospital, Shanghai Jiao Tong University School of Medicine, Shanghai, 200135 People’s Republic of China; 2 Shanghai Key Laboratory for Assisted Reproduction and Reproductive Genetics, Shanghai, People’s Republic of China; 3 Department of Obstetrics & Gynecology, Ren Ji Hospital, Shanghai Jiao Tong University School of Medicine, Shanghai, People’s Republic of China; 4 Shanghai First Maternity and Infant Hospital, Tongji University School of Medicine, Shanghai, People’s Republic of China

**Keywords:** Amnion, Parturition, Single cell transcriptome, Preterm birth, C–C motif chemokine ligand 20

## Abstract

**Background:**

The human amnion is an intrauterine tissue which is involved in the initiation of parturition. In-depth understanding of gene expression signatures of individual cell types in the amnion with respect to membrane rupture at parturition may help identify crucial initiators of parturition for the development of specific strategies to prevent preterm birth, a leading cause of perinatal mortality.

**Results:**

Six major cell types were revealed in human amnion including epithelial cells, fibroblasts and immunocytes as well as three other cell types expressing dual cell markers including epithelial/fibroblast, immune/epithelial and immune/fibroblast markers. The existence of cell types expressing these dual cell markers indicates the presence of epithelial-mesenchymal (EMT), epithelial-immune (EIT) and mesenchymal-immune (MIT) transitions in amnion at parturition. We found that the rupture zone of amnion exhibited some specific increases in subcluster proportions of immune and EMT cells related to extracellular matrix remodeling and inflammation in labor. The non-rupture zone exhibited some common changes in subcluster compositions of epithelial and fibroblast cells with the rupture zone in labor, particularly those related to oxidative stress and apoptosis in epithelial cells and zinc ion transport in fibroblasts. Moreover, we identified that C–C motif chemokine ligand 20 (CCL20) was among the top up-regulated genes in amnion epithelial cells, fibroblasts and immunocytes in the rupture zone at parturition. Studies in pregnant mice showed that administration of CCL20 induced immunocytes infiltration to tissues at the maternal–fetal interface and led to preterm birth.

**Conclusions:**

Apart from the conventional epithelial, fibroblast and immunocytes, human amnion cells may undergo EMT, EIT and FIT in preparation for parturition. Intense inflammation and ECM remodeling are present in the rupture zone, while enhanced apoptosis and oxidative stress in epithelial cells and zinc ion transport in fibroblasts are present in amnion regardless of the rupture zones at parturition. CCL20 derived from the major cell types of the amnion participates in labor onset.

**Supplementary Information:**

The online version contains supplementary material available at 10.1186/s13578-022-00797-4.

## Introduction

Preterm birth is the leading cause of perinatal morbidity and mortality [1–3]. Our incomplete understanding of the mechanisms that initiate parturition impedes the development of effective strategies for prevention of preterm birth. While activation of the fetal membranes is known to play a pivotal role in the onset of parturition [4–6], the upstream signals that provoke membrane activation remain incompletely understood. Unravelling these upstream signals may provide valuable therapeutic targets for preterm birth.

The human fetal membranes are composed of two layers of tissue, the amnion and chorion, which overlie the inner side of both the uterus body and the cervix [7]. It is believed that the fetal membranes overlying the cervix undergo the most striking physical and biochemical changes towards the end of gestation, thus becoming a source of triggers for parturition as well as the rupture point of the membranes in parturition [8, 9]. This region overlying the cervix is referred to as the zone of altered morphology (ZAM) with the region outside of ZAM, known as non-ZAM, being believed to remain largely unaltered at parturition. Several transcriptomic studies using bulk fetal membrane tissue have revealed a number of differential molecular signatures between the ZAM and non-ZAM regions at term parturition [10, 11]. However, these bulk transcriptomic studies assumed a homogeneous population of cell types, which ignores the stochasticity of gene expression in different cell types. Thus, bulk transcriptomic studies may conceal significant changes in a particular cell type, which may comprise the most important signatures of initiation of parturition. To this end, single cell RNA sequencing (scRNA-seq) is a state-of-the-art technology for identifying gene expression signatures in a particular cell type. Herein, we attempted to reveal such novel signatures associated with parturition in a single cell type with respect to ZAM and non-ZAM regions by taking advantage of scRNA-seq technology.

Although the anatomical layers of fetal membranes may vary across different species, the inner layer is consistently the amnion membrane. Accumulating evidence indicates that the amnion is not only the most tensile layer of the membranes, but is also the source of crucial signals which initiate labor and parturition [12–15]. In the amnion, surface-aligning epithelial cells and mesenchymal fibroblast cells are acknowledged to be the two major cell types, which are joined by occasional immune cells in the mesenchymal layer [7]. Emerging evidence indicates that there may be an increasing number of cells exhibiting phenotype transition such as epithelial-mesenchymal transition (EMT) in the amnion toward the end of gestation, which may become a novel source of triggers for labor [16–18]. It is now widely accepted that inflammation of the fetal membranes is an indispensable process of parturition [6]. However, apart from local in-dwelling immune cells, it remains to be determined whether amnion cells undergo any phenotype transition toward immune cells in parturition. Resolution of this issue may explain how the inflammation process is initiated in the fetal membranes in parturition.

To address the issues described above, we performed scRNA-seq on human amnion cells isolated from both ZAM and non-ZAM regions in labor, and generated a novel comprehensive transcriptomic profile of single cell signatures. Six major cell types were identified in the amnion including conventional epithelial, fibroblast and immune cells as well as cell types expressing dual cell markers including epithelial/fibroblast, epithelial/immune and fibroblast/immune markers. Although the non-ZAM region manifested some changes, the ZAM region exhibited the most pronounced changes in the proportions of cell subclusters with unique signatures of gene expression in labor. Among these signatures, the chemokine C–C motif chemokine ligand 20 (CCL20) was identified as one of the top up-regulated genes in all the major cell types at parturition. The crucial role of CCL20 in labor initiation was further validated in both human and mouse studies.

## Results

### Six cell types were identified in human amnion with scRNA-seq

**Fig. 1:**
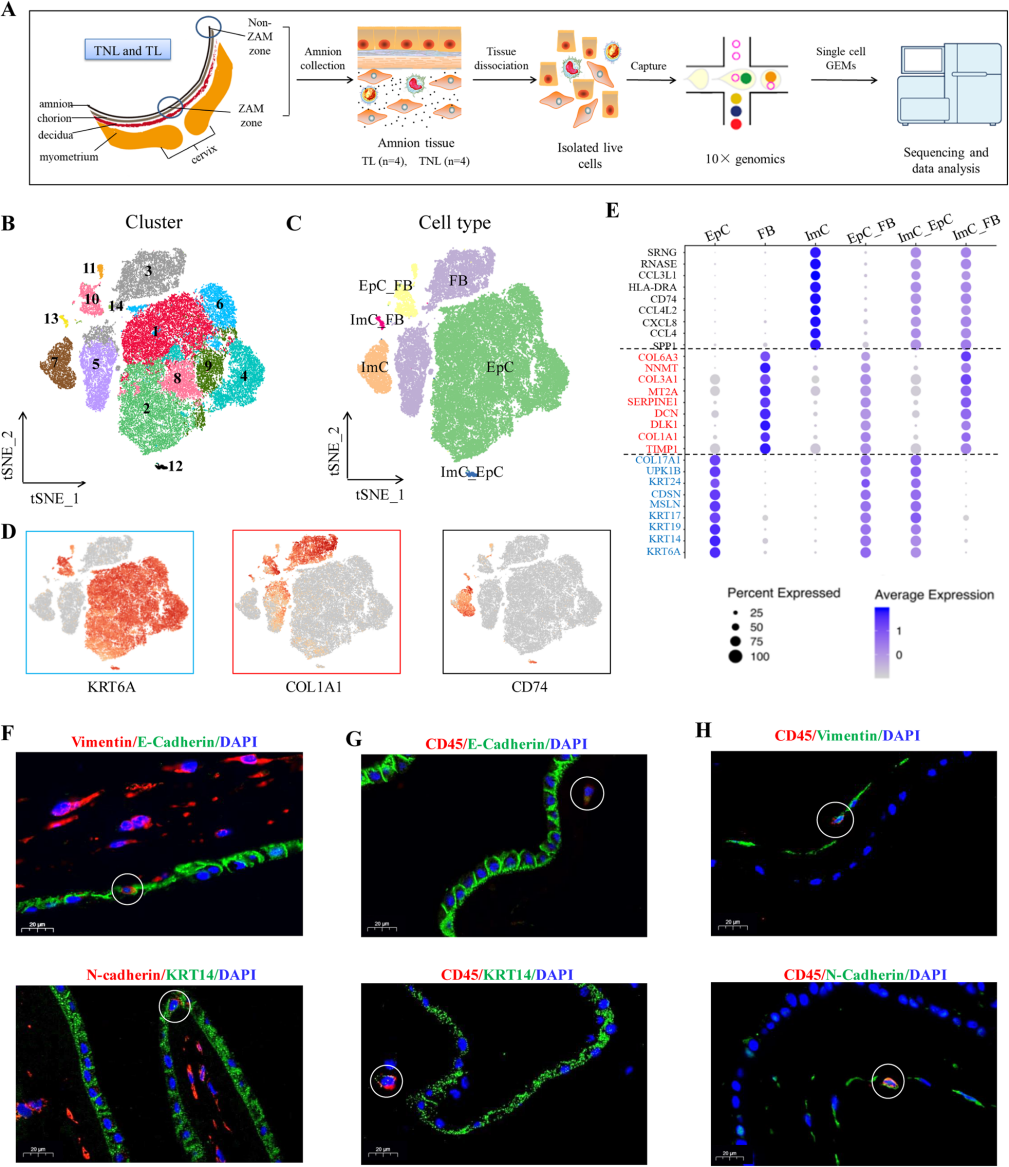
Single-cell transcriptomic atlas of the human amnion. **A** Workflow of single-cell RNA-sequencing analysis. **B**, **C** The t-distributed stochastic neighbor embedding (t-SNE) plot of 72,818 cells profiled with each cell color-coded to indicate different cell clusters (**B**) and cell types (**C**). 14 clusters and 6 major cell types were identified. FB, fibroblasts; EpC, epithelial cells; ImC, immunocytes; EpC_FB, epithelial_fibroblasts; ImC_EpC, immune_epithelial cells and ImC_FB, immune_fibroblasts. **D**, **E** Feature (**D**) and dot (**E**) plots showing the expression of established markers of epithelial cells, fibroblasts and immunocytes. Dot size encodes the percentage of cells expressing the gene, and color encodes the average level of gene expression per cell. **F** Co-staining of epithelial/fibroblast markers E-cadherin (green)/vimentin (red) or KRT14 (green)/N-cadherin (red). **G** Co-staining of epithelial/immunocyte markers E-cadherin (green)/CD45 (red) or KRT14 (green)/CD45 (red). **H** Co-staining of fibroblast/immunocyte markers vimentin (green)/CD45 (red) or N-cadherin (green)/CD45 (red). Nuclei were counterstained blue with DAPI. Scale bar, 20 μm

Fresh amnion tissue was collected from eight women who were delivered by term spontaneous labor (term labor, TL, n = 4) or term elective c section without labor (term non-labor, TNL, n = 4). Paired ZAM and non-ZAM tissues were sampled from the amnion of each woman with spontaneous labor, and designated as term labor-proximal (TL_P, n = 4) and term labor-distal (TL_D, n = 4) respectively. In the case of elective c section without labor, tissue was sampled only from the artificial rupture site over the cervix, which was designated as term non-labor-proximal (TNL_P, n = 4). Total amnion cells were isolated from the tissues sampled above for subsequent scRNA-seq using a commercial 10 × Genomics platform (Fig. 1A). After quality control, a total of 72,818 cells were captured in all these 12 samples. The number of cells captured in each sample for scRNA-seq was comparable with an average of 6081.5 ± 322.5 cells per sample (Additional file 1: Fig. S1A; Table S1). Overlaying the grouping information showed that the number of captured cells was also comparable among TNL_P, TL_P and TL_D groups (Additional file 1: Fig. S1B). Subsequent analysis of these cells from all samples with t-distributed stochastic neighbor embedding (tSNE) revealed 14 subclusters (Fig. 1B). Using established specific cell markers, 6 cell types were attributed (Fig. 1C), including epithelial cells (EpC), fibroblasts (FB), immunocytes (ImC) and three other cell types which co-expressed epithelial (*KRT6A, KRT14,* etc.)/fibroblast (*COL1A1, DCN*, etc.), epithelial (*KRT6A, KRT14,* etc.)/immune (*CD74, SRGN,* etc.) or fibroblast (*COL1A1, DCN,* etc.)/immune (*CD74, SRGN,* etc.) cell markers (Fig. 1D and 1E, Additional file 1: Fig. S2). We defined these three cell types expressing dual cell markers as epithelial_fibroblast cell (EpC_FB), immune_epithelial cell (ImC_EpC) and immune_fibroblast cell (ImC_FB) types. Additional differentially expressed genes (DEGs) of these six cell types were illustrated in the form of dot plots (Fig. 1E), heatmap (Additional file 1: Fig. S2A), feature plots (Additional file 1: Fig. S2B) and violin plots (Additional file 1: Fig. S2C). The presence of cell types expressing dual cell markers were confirmed in the amnion tissue by immunofluorescence co-staining. Cells co-expressing epithelial/fibroblast cell markers (E-cadherin/vimentin or KRT14/N-cadherin) were observed scattered in the epithelial lining of the amnion (Fig. 1F), while cells co-expressing epithelial/immune (E-cadherin/CD45 or KRT14/CD45) or fibroblast/immune (vimentin/CD45 or N-cadherin/CD45) cell markers were found sparsely distributed in the mesenchymal layer of the amnion (Fig. 1G and 1H).

Unsurprisingly, epithelial cells and fibroblasts were the two major cell types in amnion in all three groups regardless of labor status and membrane zones (Additional file 1: Table S2; Fig. S1C). Overall, the proportions of epithelial cells and fibroblasts were 67.91% and 22.04% of total cells respectively, and the other four cell types accounted for only 10.05% of total cells. The average proportions of ImC, EpC_FB, ImC_EpC and ImC_FB was 5.69%, 3.49%, 0.45%, and 0.43% of total cells respectively (Additional file 1: Table S2). The fractions of each cell types of the individual sample were given in Additional file 1: Fig. S1C. Of note, the proportions of epithelial cells and fibroblasts manifested reciprocal changes in labor with decreased proportion of epithelial cells and increased proportion of fibroblasts regardless of membrane zones, resulting in the decreased ratio of EpC/FB at both ZAM and non-ZAM with labor. The ratio of EpC/FB decreased from 5.29% in TNL_P group to 2.39% and 2.64% in TL_P and TL_D groups respectively, suggesting that there may be labor-associated phenotype transition of epithelial cells toward fibroblasts or shedding of epithelial layer. However, there were no statistical differences in the proportions of all these cell types between TL_P and TL_D or between TL_P and TNL_P. Therefore, subcluster analysis was performed subsequently for cell types with enough cell number to investigate whether there were any changes in subcluster proportions of the major cell types in association with membrane rupture at parturition.

### Subcluster analysis of the major cell types in human amnion

#### Epithelial cell

**Fig. 2:**
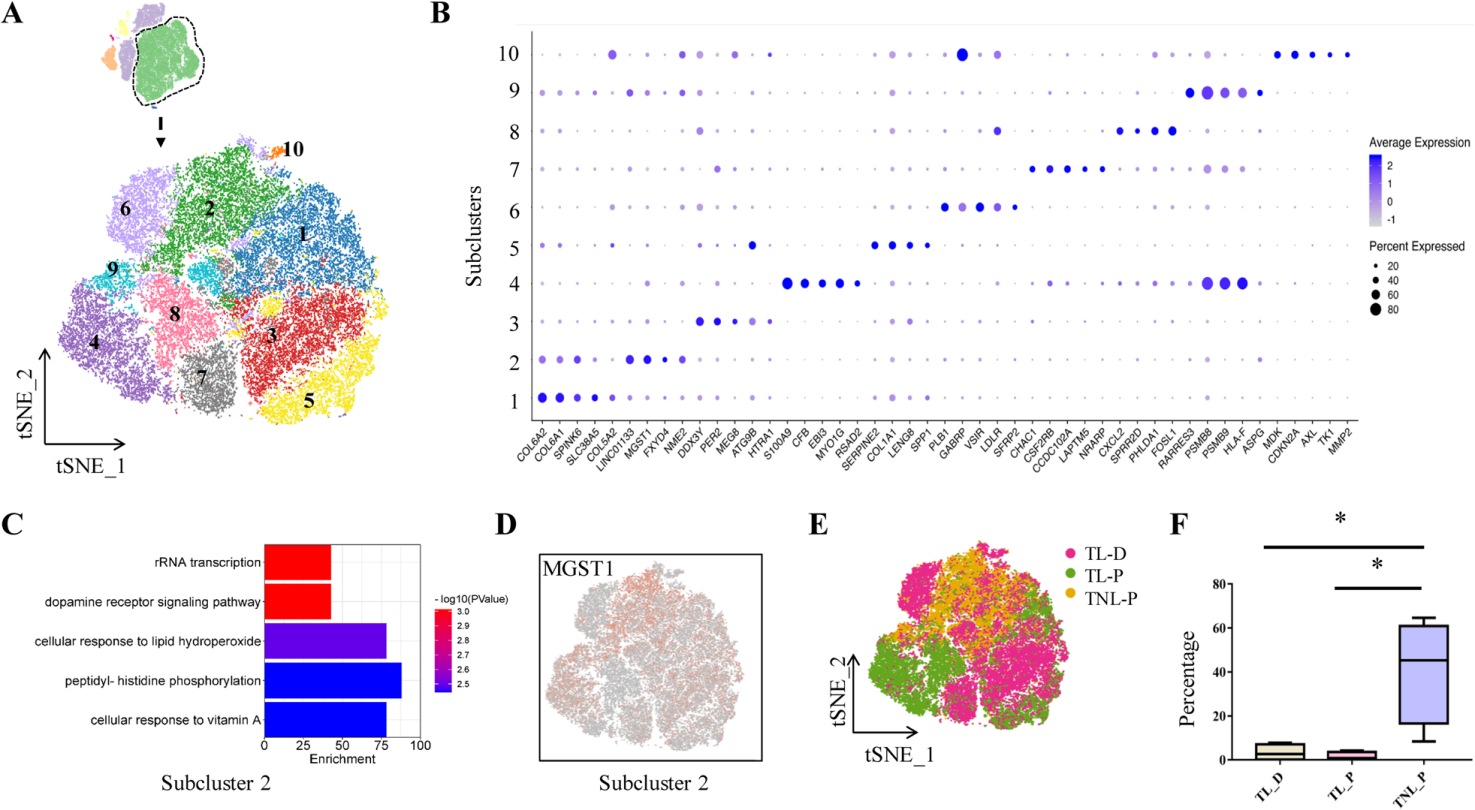
Molecular features of epithelial cell subclusters of the human amnion. **A** The t-SNE plot displays ten subclusters of epithelial cells. **B** Dot plots depict top DEGs in each subcluster of epithelial cells. **C** GO analysis of DEGs in subcluster 2. **D** Feature plot depict the expression of *MGST1* in subcluster 2. **E** Overlaying the grouping information on the t-SNE plot of epithelial cells. **F** Comparison of individual proportions of subcluster 2 in TNL_P, TL_P and TL_D groups. *P < 0.05. Unpaired Student’s t-test was used to assess the difference between TNL_P and TL_P, and paired Student’s t-test was used to assess the difference between TL_P and TL_D. *TNL_P* term non-labor-proximal, *TL_P* term labor-proximal (ZAM zone), *TL_D* term labor-distal (non-ZAM zone)

Analysis of epithelial cells of all three groups with tSNE projections revealed ten subclusters (Fig. 2A). The top DEGs of these subclusters were illustrated in dot plots (Fig. 2B). Among the ten subclusters, only the fraction of subcluster 2 manifested a significant change among the three groups. Gene ontology (GO) analysis showed that the highly expressed genes in subcluster 2 were enriched in cellular response to lipid hydroperoxide (Fig. 2C). Microsomal glutathione S-transferase 1 (*MGST1*) was among those highly expressed genes, which is known related to alleviation of oxidative stress and apoptosis [19, 20] (Fig. 2B and D). Overlaying the grouping information of the tSNE plot revealed that subcluster 2 derived mainly from the TNL_P group (Fig. 2E). Consistently, the cell number of subcluster 2 was significantly smaller in TL groups (TL_P and TL_D) than that in the TNL_P group (Fig. 2F), suggesting that enhanced oxidative stress and apoptosis may exist in amnion epithelial cells regardless of the membrane zone in labor.

#### Fibroblasts

**Fig. 3:**
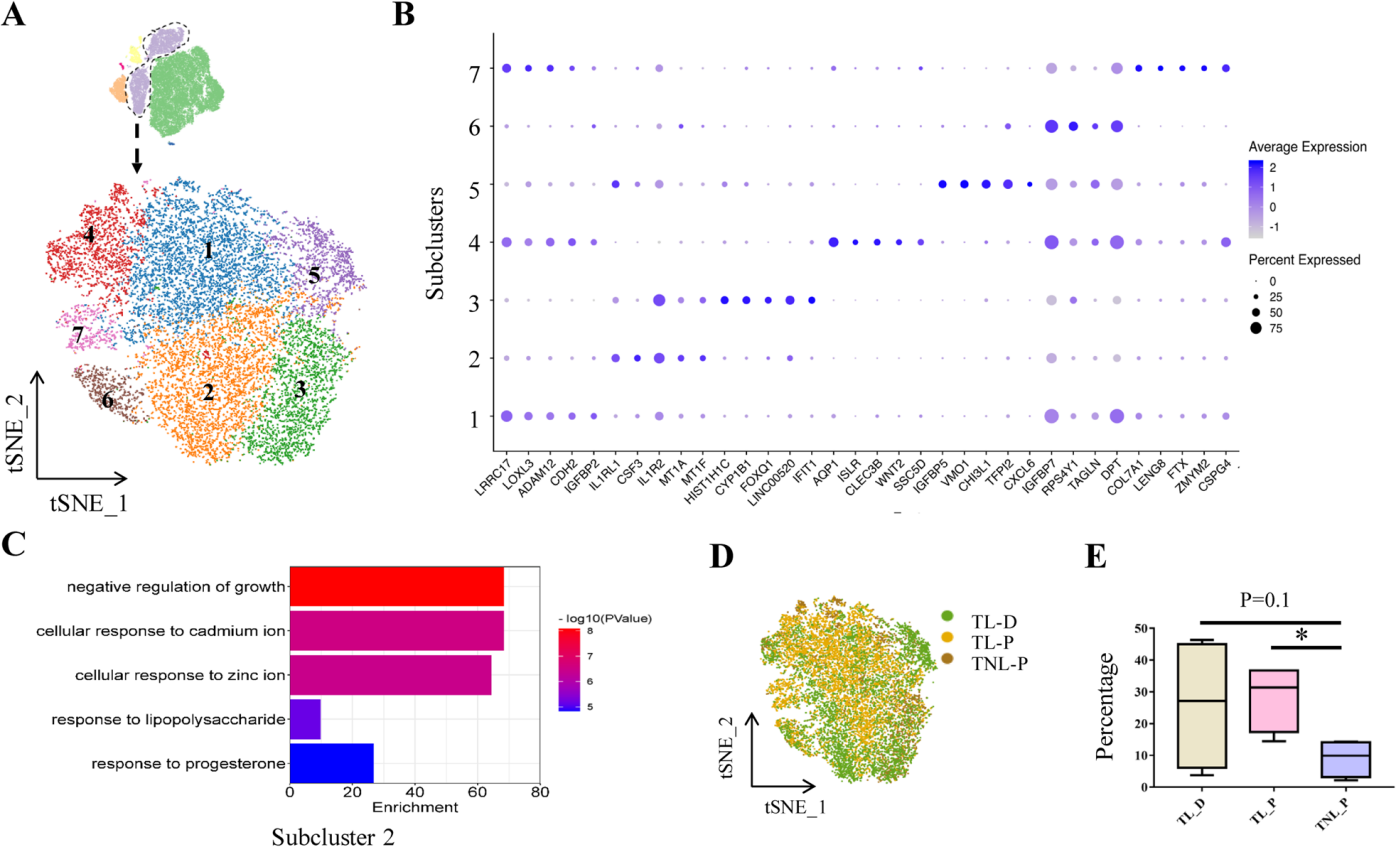
Molecular features of fibroblast subclusters of the human amnion. **A** The t-SNE plot displays seven subclusters of fibroblasts. **B** Dot plots depict top DEGs in each subcluster of fibroblasts. **C** GO analysis of DEGs in subcluster 2. **D** Overlaying the grouping information on the t-SNE plot of fibroblasts. **E** Comparison of the proportions of subcluster 2 in TNL_P, TL_P and TL_D groups. *P < 0.05. Unpaired Student’s t-test was used to assess the difference between TNL_P and TL_P, and paired Student’s t-test was used to assess the difference between TL_P and TL_D. *TNL_P* term non-labor-proximal, *TL_P* term labor-proximal (ZAM zone), *TL_D* term labor-distal (non-ZAM zone)

Analysis of fibroblasts of all three groups with tSNE projections revealed seven subclusters (Fig. 3A). The top DEGs of each subcluster were shown in dot plots (Fig. 3B). Among the seven subclusters, only the fraction of subcluster 2 manifested a significant change among the three groups. GO analysis showed that highly expressed genes in subcluster 2 were enriched in cellular responses to zinc and cadmium ions (Fig. 3C). Overlaying the grouping information of the tSNE plot revealed that subcluster 2 came mainly from TL groups (TL_P and TL_D) regardless of the membrane zone (Fig. 3D). Consistently, the proportion of subcluster 2 increased significantly in TL_P group and tended to increase in TL_D group (Fig. 3E), suggesting that zinc and cadmium ion transport might be enhanced in amnion fibroblasts in labor regardless of membrane zones.

#### Immunocyte

**Fig. 4:**
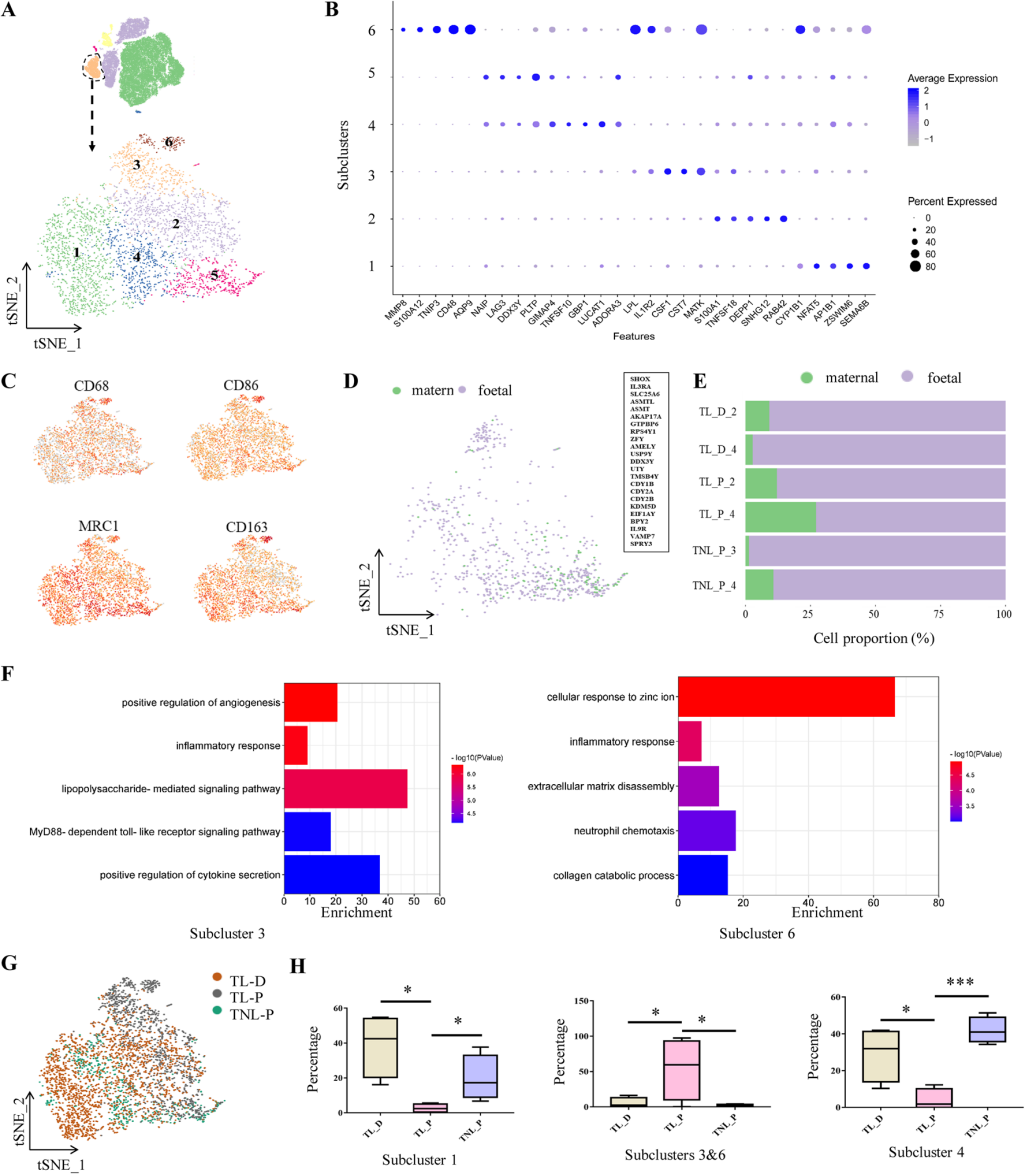
Molecular features of immunocyte subclusters of the human amnion**.** **A** The t-SNE plot displays six subclusters of immunocytes. **B** Dot plots show the top DEGs in each subset of immunocytes. **C** Feature plots of macrophage markers (*CD68, CD86, CD163* and *MRC1*). **D** The t-SNE plot displays fetal or maternal origin of immunocytes. **E** Proportions of macrophages of fetal or maternal origin in TNL_P, TL_P and TL_D groups. **F** GO analysis of DEGs in subclusters 3 and 6. **G** Overlaying the grouping information on the t-SNE plot of immunocytes. **H** Comparison of the proportions of subclusters 1, 4 and the combined proportion of subclusters 3 and 6 in TNL_P, TL_P and TL_D groups. *P < 0.05; ***P < 0.001. Unpaired Student’s t-test was used to assess the difference between TNL_P and TL_P, and paired Student’s t-test was used to assess the difference between TL_P and TL_D. *TNL_P* term non-labor-proximal, *TL_P* term labor-proximal (ZAM zone), *TL_D* term labor-distal (non-ZAM zone)

Analysis of immunocytes of all three groups with tSNE projections revealed six subclusters (Fig. 4A). The top DEGs of each subcluster were shown in the dot plots (Fig. 4B). Of note, almost all subclusters expressed macrophage-specific markers, including *CD68, CD86, CD163* and *MRC1* (Fig. 4C), and only a few cells expressed B cell marker *CD19* (Additional file 1: Fig. S3A). However, none of the cells expressed markers of T cell (*CD3*) and natural killer cell (*NCAM1*) (Additional file 1: Fig. S3B, C). These data suggest that macrophage may be the dominant immune cell in amnion. Further analysis of gene expression profiles indicated that macrophages in amnion could not be simply classified into M1 and M2 subtypes [21] because both M1 (*CD68*) and M2 (*CD163* and *MRC1*) markers were found in these macrophages despite that two other classical M1 markers, *CD80* and *NOS2,* were not detected (Additional file 1: Fig. S3D, E). To identify the origin of macrophages in amnion, male fetus datasets were analyzed to take the advantage of the unique genes encoded in Y chromosome. It turned out that the majority of macrophages expressed Y chromosome-encoded genes, suggesting that they were mostly of fetal origin rather than of maternal origin (Fig. 4D and 4E).

Among the six subclusters, only the fractions of subclusters 1, 3, 4 and 6 manifested significant changes among the three groups. GO analysis showed that the top highly-expressed genes in subclusters 1 and 4 were related to oxidative stress response and apoptotic process respectively (Additional file 1: Fig. S4). The top highly-expressed genes of both subclusters 3 and 6 were enriched in inflammatory responses, including lipopolysaccharide-mediated signaling pathway, MyD88-dependent toll-like receptor signaling pathway, positive regulation of cytokine secretion and neutrophil chemotaxis (Fig. 4F). In addition, the top highly-expressed genes in subcluster 6 were also related to cellular response to zinc ion, and extracellular matrix (ECM) disassembly (Fig. 4F), which was identified by the specifically-expressed genes including matrix metalloproteinases (*MMP1, MMP8, MMP10* and *MMP12*) and metallothioneins (*MT1A, MT1M, MT1H* and *MT1G*) (Additional file 1: Fig. S3F). In addition to *MMP*s and *MT*s, *AQP9* (aquaporin 9) was also identified among the top DEGs in subcluster 6 (Fig. 4B). Overlaying the grouping information of the tSNE plot revealed that subclusters 3 and 6 derived almost exclusively from TL_P group (Fig. 4G), whereas subclusters 1 and 4 were mostly from TL_D and TNL_P groups (Fig. 4G). Although the individual proportion of subcluster 3 or 6 showed no change, their combined proportion increased significantly in TL_P group when compared with that of TL_D group or TNL_P group (Fig. 4H). By contrast, the proportions of subclusters 1 and 4 decreased significantly in TL_P group (Fig. 4H). These data suggest that the immune cells are strengthened in metallothionein, ECM remodeling and inflammation responses, but are attenuated in apoptosis and oxidative stress in the rupture zone of the amnion in labor.

#### EpC_FB

**Fig. 5:**
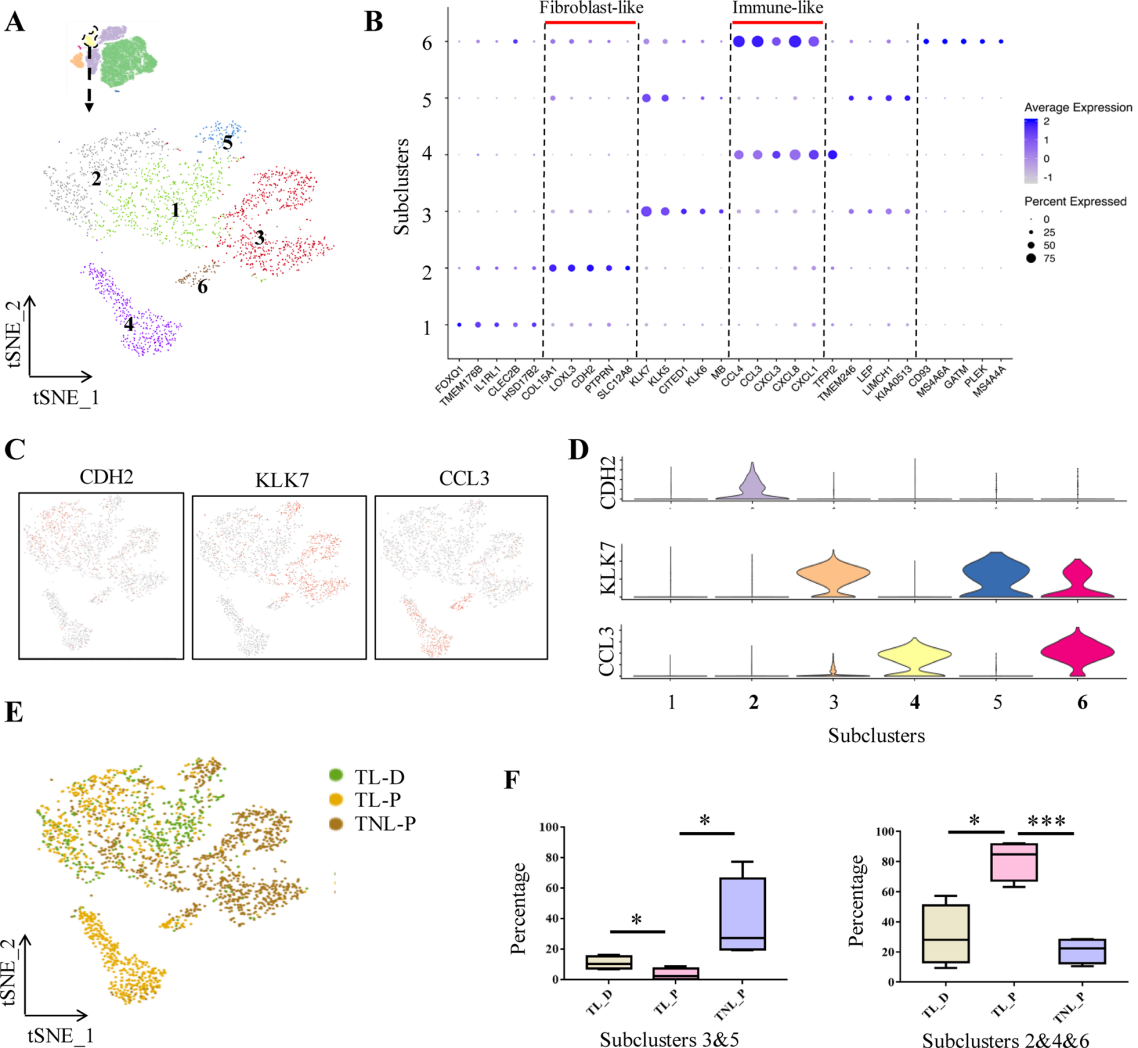
Molecular features of EpC_FB subclusters of the human amnion. **A** The t-SNE plot displays six subclusters of EpC_FB. **B** Dot plots show the top DEGs in each subset of EpC_FB. **C**, **D** Violin (**C**) and feature (**D**) plots depict *CDH2, KLK7* and *CCL3* in subclusters 2, 3, 4, 5 and 6. **E** Overlaying the grouping information on the t-SNE plot of EpC_FB. **F** Comparison of the combined proportions of subclusters 3 and 5, and the combined proportions of subclusters 2, 4 and 6 in TNL_P, TL_P and TL_D groups. *P < 0.05; ***P < 0.001. Unpaired Student’s t-test was used to assess the difference between TNL_P and TL_P, and paired Student’s t-test was used to assess the difference between TL_P and TL_D. *TNL_P* term non-labor-proximal, *TL_P* term labor-proximal (ZAM zone), *TL_D* term labor-distal (non-ZAM zone)

Analysis of EpC_FB cells of all three groups with tSNE projections revealed six subclusters (Fig. 5A). The top DEGs of these subclusters were shown in dot plots (Fig. 5B). Although there were no obvious specific marker genes in subcluster 1 (Fig. 5B), the other subclusters could be identified by specific marker genes. Subcluster 2 expressed fibroblast maker genes *CDH2* and *LOXL3,* etc. (Fig. 5B–D, Additional file 1: Fig. S5). Subclusters 4 and 6 shared the same immunocyte marker genes *CCL3* and *CCL4L2,* etc. (Fig. 5B–D, Additional file 1: Fig. S5), while subclusters 3 and 5 shared the same marker genes *KLK5, 6* and *7*, which belong to the kallikrein (*KLK*) family (Fig. 5B–D, Additional file 1: Fig. S5).

Overlaying the grouping information of the tSNE plot revealed that the fibroblast-like subcluster 2 and immunocyte-like subclusters 4 and 6 derived mostly from TL_P group, whereas *KLK*-expressing subclusters 3 and 5 derived mainly from TNL_P group (Fig. 5E). Although the individual proportion of subclusters 2, 4 and 6 did not change significantly, their combined proportion increased significantly in TL_P group (Fig. 5F) when compared with that of TL_D group or TNL_P group. By contrast, the combined proportion of subclusters 3 and 5 decreased significantly in TL_P group when compared with TL_D or TNL_P group (Fig. 5F). These data suggest that EpC_FB cells may behave in a way more like immunocyte and fibroblast with strengthened functions in inflammation and ECM remodeling in the rupture zone of the amnion in labor. However, we are unclear about the implication of decreased proportions of subclusters 3 and 5 in the rupture zone at parturition.

### Identification of crucial signatures of membrane rupture at parturition

**Fig. 6:**
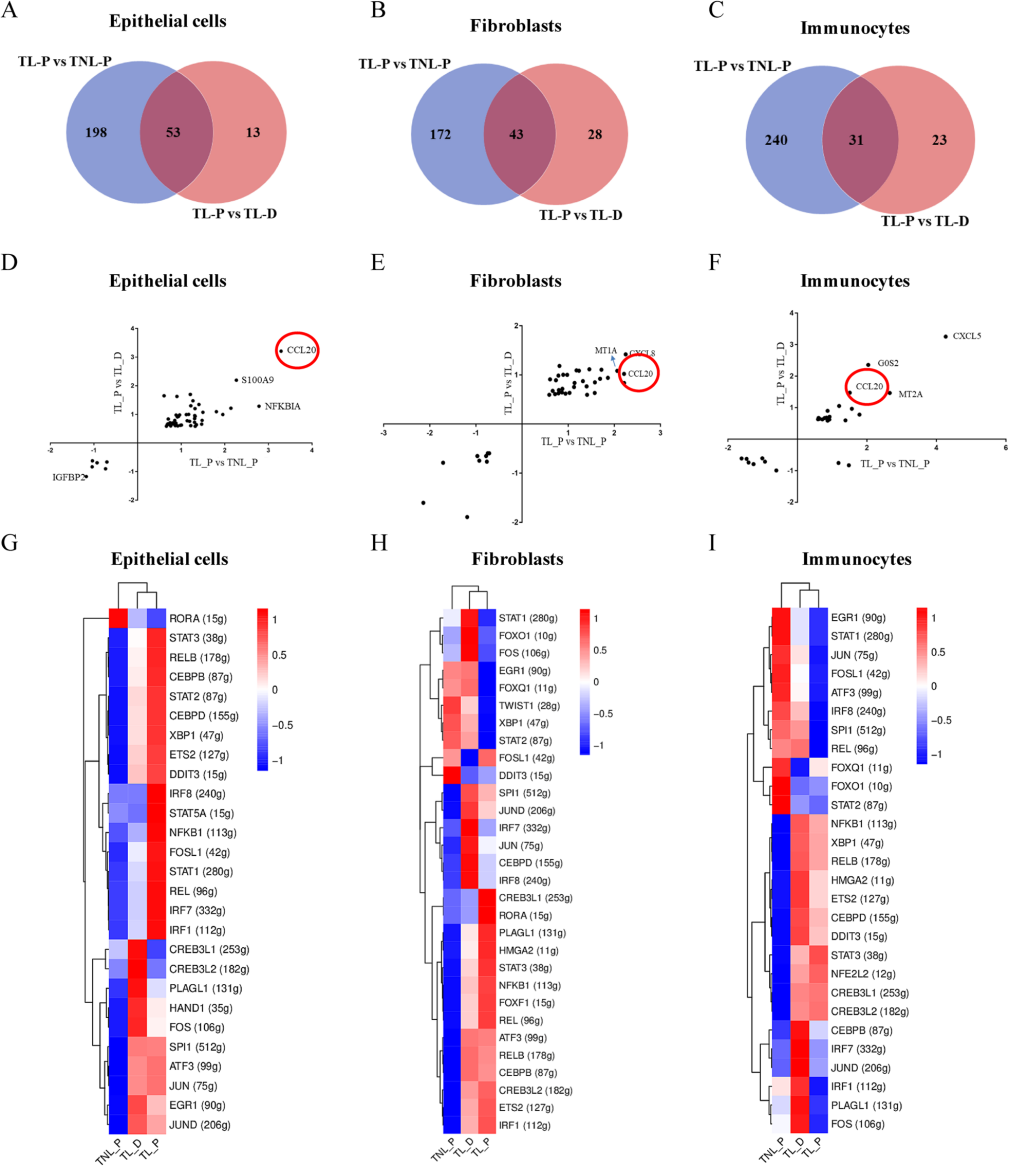
Identification of unique signatures in major cell types of human amnion in membrane rupture at parturition. **A**–**C** The Venn diagram shows overlap in DEGs (≥ 1.5-fold changes, P < 0.05) between TL_P and TNL_P groups, and between TL_P and TL_D groups in epithelial cells (**A**), fibroblasts (**B**) and immunocytes (**C**). **D**–**F** CCL20 was revealed among the top up-regulated genes in epithelial cells (**D**), fibroblasts (**E**) and immunocytes (**F**) in TL_P group. **G**–**I** Inferred active transcription factors (TFs) across TL_P, TL_D and TNL_P groups in epithelial cells (G), fibroblasts (**H**) and immunocytes (**I**) by SCENIC analysis. Numbers in brackets indicate the regulons subject to respective TFs. *TNL_P* term non-labor-proximal, *TL_P* term labor-proximal (ZAM zone), *TL_D* term labor-distal (non-ZAM zone)

To identify crucial signatures of membrane rupture at parturition, DEGs of TL_P group were compared with that of TNL_P or TL_D group in epithelial cells, fibroblasts and immunocytes, the three major cell types of the amnion. There were 251, 215 and 271 DEGs (fold change > 1.5 and p-value < 0.05) in epithelial cells, fibroblasts and immunocytes respectively between TL_P and TNL_P groups, and there were 66, 71 and 54 DEGs in epithelial cells, fibroblasts and immunocytes respectively between TL_P and TL_D groups (Fig. 6A–C, Additional file 2: Dataset 1–6). Intersection of DEGs between TL_P and TNL_P groups with DEGs between TL_P and TL_D groups revealed that there were 53, 43 and 31 genes commonly changed in epithelial cells, fibroblasts and immunocytes respectively in TL_P group (Fig. 6A–C, Additional file 2: Dataset 7–9). The specific DEGs in epithelial cells, fibroblasts and immunocytes were displayed in Additional file 2: Dataset 10–12 respectively. Of the commonly-changed genes, *CCL20* was the only one among the top up-regulated genes in epithelial cells, fibroblasts and immunocytes in TL_P group (Fig. 6D–F).

To identify the crucial transcriptional regulatory network in labor initiation, we performed single-cell regulatory network inference and clustering (SCENIC) analyses in human epithelial cells, fibroblasts and immunocytes [22]. Our data showed that the highly active transcription factors (TFs) varied specifically in TL_D, TL_P and TNL_P groups (Fig. 6G–I). Unsurprisingly, *NFKB1* gene which encodes the subunit p65 of the proinflammatory transcription factor nuclear factor-kappa B (NF-κB) presented high transcriptional activity in all the three major cell types in the TL_P group (Fig. 6G–I). In addition to *NFKB1, CEBPD* encoding CCAAT enhancer binding protein delta and *STAT3* encoding signal transducer and activator of transcription 3 were also found to be highly active in all the three major cell types in TL_P group (Fig. 6G–I), which were consistent with our previous work showing that CEBPD and STAT3 are involved in the upregulation of parturition-pertinent genes in the amnion [13, 23, 24]. By contrast, *EGR1* encoding early growth response 1, and *STAT1* encoding signal transducer and activator of transcription 1 presented low activities in both fibroblasts and immunocytes, while *RORA* encoding RAR related orphan receptor A exhibited low activity in epithelial cells in TL_P group (Fig. 6G–I).

### Verification of the crucial role of CCL20 in labor onset

**Fig. 7:**
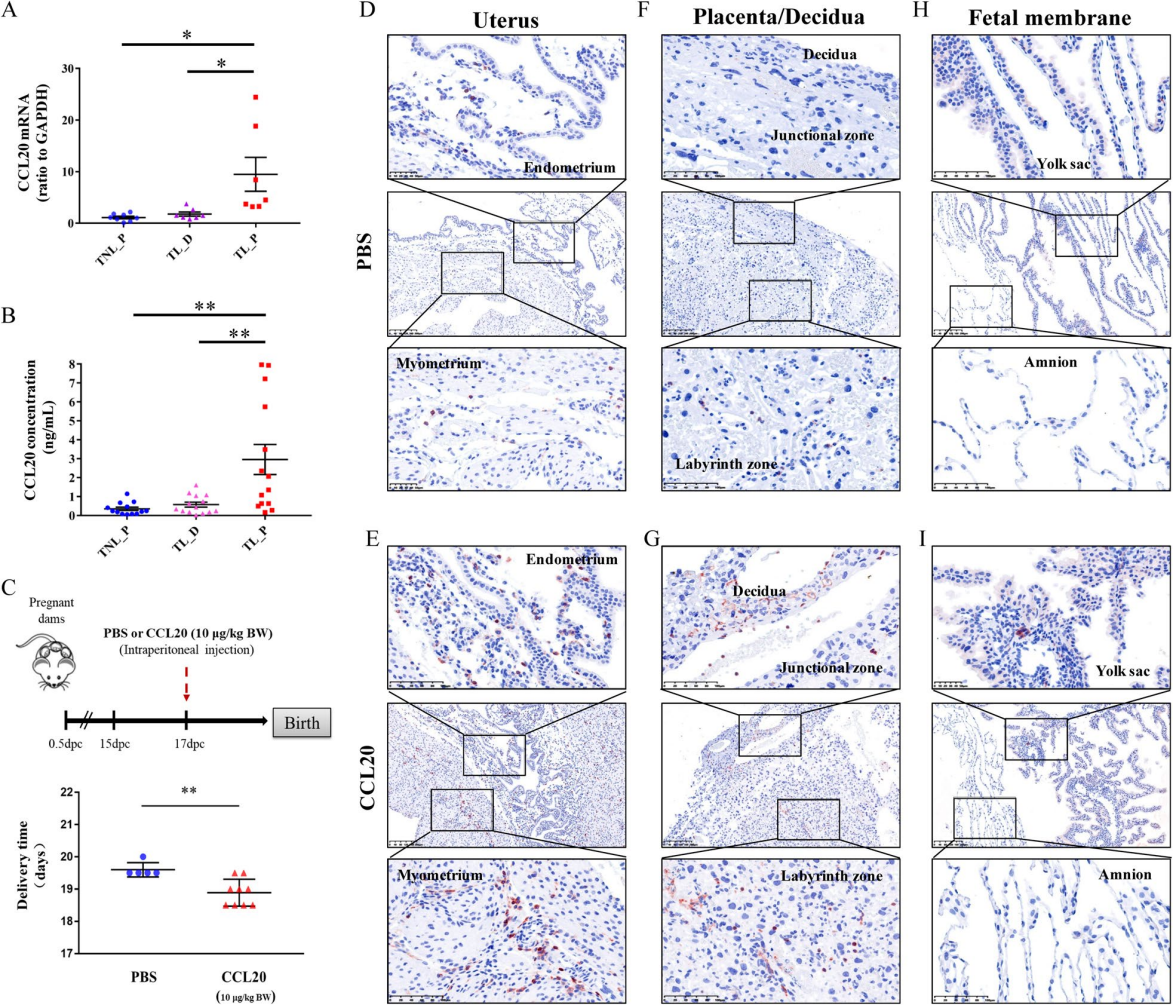
Role of CCL20 in the onset of labor. **A**, **B** Increased CCL20 mRNA (**A**) and protein (**B**) in the human amnion in TL_P group. *P < 0.05; **P < 0.01. Unpaired Student’s t-test was used to assess the difference between TNL_P and TL_P, and paired Student’s t-test was used to assess the difference between TL_P and TL_D. TNL_P, term non-labor-proximal; TL_P, term labor-proximal (ZAM zone); TL_D, term labor-distal (non-ZAM zone). **C** Intraperitoneal injection of CCL20 (10 μg/kg BW) into pregnant mice on dpc 17 induced preterm birth. **P < 0.01. Data was analyzed with unpaired Student’s t-test. **D**–**I** Immunohistochemical staining of CD45 positive cells in uterus (**D** and **E**), placenta (**F** and **G**), decidua (**F** and **G**) and fetal membranes (**H** and **I**) obtained from the mice with CCL20 or vehicle administration. Negative control was shown in Additional file 1: Fig. S6

The increase in CCL20 in the amnion in TL_P group was verified by measurements of *CCL20* mRNA with quantitative real time PCR (qRT-PCR) (Fig. 7A), and CCL20 protein with enzyme-linked immunosorbent assay (ELISA) (Fig. 7B) and immunohistochemical staining (Additional file 1: Fig. S6) in human amnion tissue. Studies in the mouse demonstrated that intraperitoneal injection of CCL20 (10 μg/kg body weight (BW)) at 17 dpc induced preterm birth by 0.5 to 1 day with no fetal demise (Fig. 7C). Histological examination of cells expressing CD45, a unique membrane glycoprotein marker, which is expressed in almost all hematopoietic cells except mature erythrocytes, in intrauterine tissues revealed an increased number of CD45-positive cells in endometrium and myometrium of the uterus (Fig. 7D and 7E), the junctional and labyrinth zones of the placenta (Fig. 7F and 7G), and the decidua (Fig. 7F and 7G) in animals receiving CCL20 administration, suggesting increased immune cell infiltration into these intrauterine tissues. However, only a few immunocytes were observed in the yolk sac membrane and none were observed in the amnion of the fetal membranes in animals receiving CCL20 administration (Fig. 7H and 7I), suggesting that the yolk sac membrane but not the amnion is the site of immune cell infiltration although it is not as great as in the other intrauterine tissues following CCL20 administration.

## Discussion

Single cell RNA-seq has been applied to interrogate cellular heterogeneity and inter-cellular communications at the maternal–fetal interface in normal pregnancy [25–30] as well as in pregnancy disorders such as recurrent miscarriage [31, 32], preeclampsia [33], gestational diabetes mellitus [34] and preterm birth [35, 36]. A pioneering study of preterm birth by Roger Pique-Regi et al*.* [36] analyzed maternal and fetal cells in major tissues at the maternal–fetal interface, including placental villous tree, basal plate and chorioamniotic membranes with scRNA-seq. Their study provided a catalogue of cell types and transcriptional profiles relevant to term and preterm parturition in each of the compartments. However, in the case of chorioamniotic membranes, they used whole membrane layers with attached decidua without distinguishing the ZAM and non-ZAM regions. Therefore, it is not clear in which layer of the fetal membranes the identified cell types dwell in their study. The relationship of these identified cell types with the rupture zone is also not clear. In addition, cell types isolated from whole membranes with attached decidua are far more complex than cell types isolated from a single layer, and may therefore overlook some minor cell types in a particular layer. Given the crucial importance of the amnion for the maintenance of membrane tensile strength and generation of parturition signals [4, 6], single cell analysis of the amnion layer with respect to the altered zones of morphology is thus of utmost importance for resolving the mechanism of membrane rupture at parturition.

The amnion is mainly composed of epithelial cells, fibroblasts [7], and to a lesser extent, immune cells. In addition to these three cell types, here we demonstrated additional three cell types expressing dual cell markers, i.e. EpC_FB, ImC_FB and ImC_EpC, in amnion, which suggests that there may be phenotype transitions from epithelial cells to mesenchymal cells (EMT), from epithelial cells to immunocytes (EIT) and from fibroblasts to immunocytes (MIT) in amnion toward the end of gestation. EMT has been shown to participate in a number of physiological and pathological processes [16–18, 37, 38]. Increasing EMT has been reported in the amnion toward the end of gestation in preparation for membrane rupture and labor [16–18]. Although much less is known about MIT, Kim et al*.* found the existence of cells expressing both fibroblast and macrophage markers in chorioamniotic membranes in labor by using immunofluorescence staining [39]. Recently, EIT has been observed in cancers [40–42]. Our study demonstrated for the first time that EIT might also exist in amnion. The existence of cell types expressing dual cell markers indicate that amnion cells may undergo phenotype transitions toward fibroblast and immune cells in favor of inflammation and ECM remodeling in preparation for membrane rupture and parturition.

Although there were no significant changes in the proportions of the six major cell clusters, the subcluster proportions of the four major clusters including EpC, FB, ImC and EpC_FB, which have enough cells for subcluster analysis, displayed significant changes in amnion at parturition either regardless of the rupture zone or specifically in the rupture zone. We found that the subcluster proportion of EpC in association with apoptosis and oxidative stress alleviation decreased, while the subcluster proportion of FB in association with zinc and cadmium ion transport increased in amnion at parturition regardless of the rupture zone, suggesting that the non-rupture zone of the amnion was not inert in labor. At least, oxidative stress and apoptosis in epithelial cells and zinc ion transport in fibroblasts are enhanced in this region together with the rupture region in labor. It has been reported that oxidative stress and apoptosis in amnion epithelial cells may account for cell senescence and shedding of epithelial cells as well as inflammation of the membranes in labor [43–46]. Zinc ion is required by a number of proteases for the proteolytic activity [47, 48]. Thus, increased zinc ion transport in fibroblasts may facilitate protease activity for ECM remodeling in amnion at parturition.

In contrast to EpC and FB, the subcluster proportions of ImC and EpC_FB appeared to change specifically in the rupture zone of the amnion in parturition. We found that the subcluster proportions of ImC related to zinc ion transport, ECM disassembly and inflammatory response increased while the subcluster related to apoptosis and oxidative stress response decreased. The increase in subcluster related to inflammation is in line with previous reports that the rupture site of the membranes is the principle region of inflammation during parturition [11, 49]. Decreases in ImC subcluster related to apoptosis and oxidative stress responses may save immune cells for functions of inflammation and ECM remodeling. However, we do not have a clue why the proportion of EpC_FB subclusters expressing *KLKs* decreased in the rupture zone of the amnion at parturition, given the serine protease property of kallikreins encoded by *KLKs* [50].

It is now widely accepted that parturition is an inflammatory process [51], whereby a coalition of leukocytes infiltrate the fetal-maternal interface, including the uterus, decidua and chorioamniotic membranes [52–54]. Although the amnion had been reported to exhibit leukocyte chemotactic activity during labor [55], very few leukocytes have been observed in the amnion in previous studies [56, 57], suggesting that other tissues at the maternal–fetal interface are the principle sites of immune cell infiltration in labor. In this study, we found that a substantial number of immune cells were present in amnion, but interestingly, almost all these immunocytes are macrophage-like cells of fetal origin, suggesting that they are either locally in-dwelling macrophages or macrophages migrating from the fetal side. Of interest, we found that macrophages in the amnion could not be simply divided into M1 and M2 subtypes, and they expressed a coalition of M1 and M2 makers. It is generally believed that M2 macrophages can produce abundant MMPs and are involved in homeostasis and tissue remodeling, while M1 macrophages play a role in Th1-type immune responses [58, 59]. Because the amnion is the most tensile layer of the membranes, the M2 phenotype of macrophages may assist in membrane rupture by remodeling ECM of the amnion in labor [60] and the M1 phenotype may assist in the inflammatory reactions of the amnion in labor. These properties of M1 and M2 macrophage subtypes may explain why the amnion contains macrophages expressing both M1 and M2 markers. In addition, we also observed a few cells expressing B cell but not T cell or NK cell markers in the amnion. These observations suggest that the amnion may also be a site of immune cell infiltration though to a lesser extent.

Increased CCL20 abundance in the amniotic fluid has been reported in term and preterm labor [61, 62]. In the present study, we found that CCL20 increased dramatically in the rupture zone of the amnion in labor and we identified *CCL20* as one of the top up-regulated genes in all the major cell types of the amnion including epithelial cells, fibroblasts and immunocytes in this zone in labor. Studies in the mouse showed that intraperitoneal injection of CCL20 induced preterm labor, which indicates a crucial role of CCL20 in labor onset. CCL20 is the only chemokine known to interact with CC chemokine receptor 6 (CCR6). The ligand-receptor pair CCL20-CCR6 is responsible for the chemoattraction of immature dendritic cells, effector/memory T-cells and B-cells and plays a role in homeostatic and inflammatory conditions [63]. In this study, we observed CD45-positive cells in the uterus, placenta, decidua and yolk sac membrane but not the amnion following injection of CCL20 into the pregnant mouse, suggesting that CCL20 facilitates the infiltration of immune cells mainly into the uterus, placenta, decidua and yolk sac but not the amnion. This situation likens the situation of human parturition, in which infiltration of immune cells has been shown mainly to the uterus, placenta, decidua and chorion but not the amnion [52–54].

**Fig. 8:**
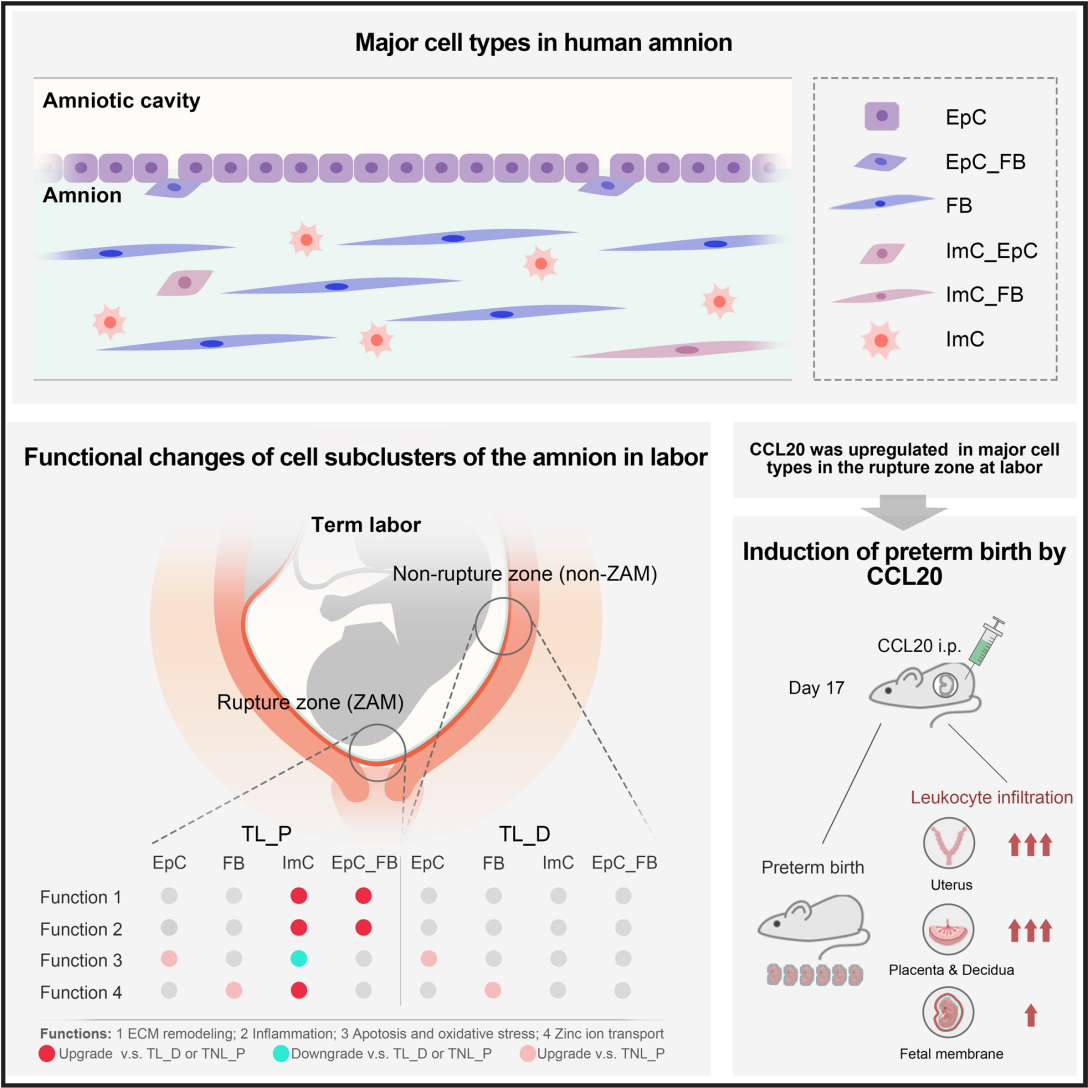
Diagram illustrating the main findings of this study. Six major cell types were identified in the human amnion including EpC, FB, ImC, EpC_FB, ImC_EpC and ImC_FB. The membrane rupture zone (ZAM) exhibited specific changes in subcluster proportions of ImC and EpC_FB in labor, particularly those subclusters related to ECM remodeling and inflammation. Additionally, the non-rupture zone also manifested some common changes in EpC and FB subcluster compositions along with the rupture zone, particularly those subclusters related to oxidative stress and apoptosis in EpC and zinc ion transport in FB. CCL20 was upregulated in the major cell types of the amnion in the rupture zone at parturition. Administration of CCL20 induced immune cell infiltration to intrauterine tissues and led to preterm birth in the mouse. i.p., intraperitoneal injection. *FB* fibroblasts, *EpC* epithelial cells, *ImC* immunocytes, *EpC_FB* epithelial_fibroblasts, *ImC_EpC* immune_epithelial cells, *ImC_FB* immune_fibroblasts

In conclusion, we have identified six major cell types in the human amnion including epithelial cells, fibroblasts, immunocytes, EpC_FB, ImC_EpC and ImC_FB (Fig. 8). The existence of EpC_FB, ImC_EpC and ImC_FB cell types indicates the presence of EMT, EIT and MIT in the amnion in preparation for parturition. We found that the membrane rupture zone exhibited some specific changes in subcluster proportions of ImC and EpC_FB in labor, particularly those subclusters related to ECM remodeling and inflammation (Fig. 8). We found that the non-rupture zone also manifested some common changes in EpC and FB subcluster compositions along with the rupture zone, particularly those subclusters related to oxidative stress and apoptosis in EpC and zinc ion transport in FB (Fig. 8). Moreover, we identified that CCL20 was one of the top up-regulated genes in the major cell types of the amnion including epithelial cells, fibroblasts and immune cells in the rupture zone at parturition. Increased CCL20 expression was confirmed in the amnion obtained from pregnant women with labor. Indeed, a murine study showed that administration of CCL20 induced preterm birth with immune cell infiltration to tissues at the maternal–fetal interface (Fig. 8).

## Materials and methods

### Study population

Women with singleton pregnancies were recruited between 1 September 2019 and 1 October 2020 under a protocol approved by the Ethics Committee of Ren Ji Hospital, Shanghai Jiao Tong University School of Medicine with written informed consent (Protocol No. [2013] N025). Pregnancies with complications including preeclampsia, gestational diabetes, fetal growth restriction, and chorioamnionitis were excluded from this study. For scRNA-seq, amnion tissue samples were collected from four women with spontaneous labor at term (term labor, TL) and four women undergoing elective c section without labor at term (term non-labor, TNL). In the case of TL, paired amnion tissues were sampled from the ZAM [designated as term labor-proximal (TL_P)] over the cervix and non-ZAM [designated as term labor-distal (TL_D)], 10 cm distal to the ZAM. In the case of TNL, amnion tissue was sampled at the artificial rupture site over the cervix, which was designated as term non-labor-proximal (TNL_P). For validation of the changes of crucial gene expression in amnion in parturition observed with scRNA-seq, another twenty-eight pregnant women were recruited into TL and TNL groups for analysis with qRT-PCR and ELISA. The number of pregnant women was given in the figure legend of each of the studies. The clinical information for all subjects is given in Additional file 1: Table S3.

### Preparation of human amnion single-cell suspensions for library generation and sequencing

Fresh amnion was minced into small pieces and digested twice with 0.125% trypsin (Life Technologies Inc., Grand Island, NY) followed by further digestion with 0.1% collagenase (Sigma, St. Louis, MO). All digestions were combined and centrifuged for collection of amnion cells. To avoid any cell aggregates from the single-cell suspension, the cell suspension was filtered through a 40-μm nylon mesh. Collected cells were washed and resuspended in calcium/magnesium-free phosphate-buffered saline (PBS) containing 0.1% bovine serum albumin. Cell viability was assessed by trypan blue (Bio-Rad, Irvine, CA) staining. Cell viability ≥ 90% was used for library generation on 10 × Genomics system. Briefly, the cells were partitioned for genetically engineered model (GEM) generation and barcoded cDNA library construction using 10 × Chromium Single-cell kits following the manufacturer’s protocol. Then all libraries were subjected to quality tests on a Fragment Analyzer 2100 (Agilent Technologies, Carpinteria, CA) and sequenced on the Illumina sequencing platform (NovaSeq 6000; Illumina, San Diego, CA).

### Quality control of scRNA-seq data and identification of cell clusters and subclusters

Raw sequencing data were processed using the Cell Ranger software pipeline (version 3.1.0) provided by 10 × Genomics. Raw binary base call files were demultiplexed using the *cellranger mkfastq*. Subsequent read alignments and transcript counting were performed individually for each sample using *cellranger* counts with standard parameters. Then, unique molecular identifier (UMI) count matrix was imported into the Seurat R package (version 3.2.0) [64]. Quality control of each constructed library was performed. A criterion was applied to filter out cells with genes fewer than 500 or a mitochondrial gene ratio greater than 10% or UMI/gene numbers out of the limit of mean value ± two fold of standard deviations assuming a Guassian distribution of each cells' UMI/gene numbers. Moreover, the batch effects on single-cell RNA-sequencing data were removed by analyzing the mutual nearest neighbors (MNN) with the R package ‘batchelor’ [65].

After quality standardization, the Seurat R package was applied to analyze the scRNA-seq data [66]. Firstly, library size normalization was performed with Seurat on the filtered matrix to obtain the normalized count, and then top variable genes across single cells were identified. After that, principal component analysis (PCA) was performed to reduce the dimensionality on the log-transformed gene-barcode matrices of top variable genes. Cells were clustered based on a graph-based clustering approach (Find Clusters function), and were visualized in 2-dimension using tSNE. FindAllMarkers function (test.use = “bimod”) in Seurat was used to identify marker genes of each cluster. For a given cluster, positive markers were identified by the FindAllMarkers function compared with all other cells. GO analyses were conducted using R based on the hypergeometric distribution.

For subcluster analysis, cell types with cell number large enough were extracted via the “SubsetData” function following primary annotation. “FindClusters” and “FindAllMarkers” functions were applied, and the selected cells were reclustered by tSNE and annotated by the dominant cell markers.

### Verification of cell types expressing dual cell markers in human amnion with immunofluorescence staining

To confirm the existence of cell types expressing dual cell markers observed with scRNA-seq, immunofluorescence co-staining of the amnion tissue was carried out. Amnion tissue was collected from elective c section without labor at term and fixed with 10% neutral buffered formalin at room temperature for 24 h. The tissue was then transferred to 70% ethanol, followed by paraffin embedding. Paraffin-embedded amnion tissue was sectioned at 5 μm thickness. After deparaffination, the section was permeabilized with 0.4% Triton X-100. After blocking with normal serum, the section was incubated with the combination of two different primary antibodies representing different cell types overnight at 4 °C, followed by incubation with respective Alexa Fluor 488- and 594-conjugated secondary antibodies (Life Technologies) at 37 °C for 2 h. Primary antibodies used for immunofluorescence co-staining are as follows: immune cell markers: rabbit anti-CD45 (1:100; Cell Signaling, Danvers, MA; 13917S); mesenchymal cell markers: mouse anti-vimentin (1:200; NOVUS, Centennial, CO; NBP1-92687), rabbit anti-vimentin (1:200; Proteintech, Wuhan, China; 10366-1), mouse anti-N-cadherin (1:100; Cell Signaling; 14215S); epithelial cell markers: rabbit anti-E-cadherin (1:200; Cell signaling; 3195S), mouse anti-E-cadherin (1:200; Abcam, Cambridge, MA; ab1416), mouse anti-cytokeratin 14 (KRT14) (1:200; Proteintech; 60320-1). Nuclei were counterstained with 4’,6-diamidino-2-phenylindole (DAPI; 1 μg/mL; blue color). The slides were examined under a fluorescence microscope (Zeiss, Oberkochen, Germany).

### Analysis of immune cell origin in human amnion

To analyze the origin of immune cells detected in the amnion with scRNA-seq, samples from only male fetuses were analyzed to take the advantage of the unique genes (*DDX3Y, USP9Y EIFAY, *etc*.*) encoded in the Y chromosome of the male fetus. Gene expression matrices were used to perform Seurat alignment and t-SNE clustering as described above. Detection of any expression of the Y chromosome–encoded genes indicated fetal origin, otherwise an indication of maternal origin.

### Identification of unique signatures in major cell types of human amnion in membrane rupture at parturition

To identify unique parturition-pertinent signatures in the major cell types of human amnion in different groups, DEGs of TL_P vs TL_D or TL_P vs TNL_P were identified using the FindMarkers function (test use = “MAST”) in Seurat package [67]. P value < 0.05 and |log2foldchange|> 0.58 were set as the significant threshold. DEG’s GO analyses were conducted using R based on the hypergeometric distribution.

The active TFs in the major cell types of human amnion in different groups were analyzed with SCENIC analysis utilizing the motifs database for RcisTarget and GRNboost [22]. Briefly, over-represented TF binding motifs on a gene list were identified with the RcisTarget package. The AUCell package was used to score the activity of regulons in each single cell of each group. More detailed descriptions of SCENIC analysis are available on line at https://github.com/aertslab/SCENIC.

### Measurement of CCL20 in human amnion with qRT-PCR, ELISA and immumohistochemical staining

To confirm the changes of the signature molecule CCL20 observed with scRNA-seq in amnion at the rupture site with labor, qRT-PCR, ELISA and immumohistochemical staining were conducted.

For qRT-PCR and ELISA, RNA and protein were extracted from amnion tissue obtained from twenty-eight pregnant women with and without labor. Briefly, amnion tissue was cut within or 10 cm distal to the spontaneous rupture site with labor and from the artificial rupture site without labor, and then grounded in liquid nitrogen. For *CCL20* mRNA measurement, total RNA was extracted from the homogenized ground tissue using total RNA isolation kit (Foregene, Chengdu, China). After examination of RNA quality, reverse transcription was carried out using a Prime-Script RT Master Mix Perfect Real Time Kit (TaKaRa, Kyoto, Japan). The amount of *CCL20* mRNA was determined with qRT-PCR using the above reverse-transcribed cDNA and power SYBR® Premix Ex Taq™ (TaKaRa) following a previously described protocol [68]. Housekeeping gene glyceraldehyde 3-phosphate dehydrogenase (GAPDH) was measured in parallel for normalization. The relative mRNA abundance was quantitated using the 2−^∆∆Ct^ method. Primer sequences for PCR were as follows: *GAPDH*, forward, 5’-CCCCTCTGCTGATGCCCCCA-3’ and reverse, 5’-TGACCTTGGCCAGGGGTGCT-3’; *CCL20*, forward, 5’- TCCTGGCTGCTTTGATGTCA-3’ and reverse 5’- CAAAGTTGCTTGCTGCTTCTGA-3’. For CCL20 protein measurement, grounded tissue was homogenized and lysed in ice-cold RIPA lysis buffer containing a protease inhibitor cocktail. After centrifugation, the supernatant was collected for measurement of CCL20 protein with an ELISA kit (R&D Systems, Minneapolis, MN; DM3A00) following the protocol provided by the manufacturer.

For immumohistochemical staining, the amnion tissue collected from deliveries with and without labor at term was used. The endogenous peroxidase activity of the tissue section was quenched with 0.3% H_2_O_2_ following deparaffination. After blocking with normal serum, the section was incubated with a primary antibody against CCL20 (Novus; AF360) at 1:100 dilution or non-immune serum for negative control overnight at 4◦C. After washing with PBS, the section was incubated with a biotinylated secondary antibody for 2 h. After washing, the avidin–biotin complex reagent conjugated with horseradish peroxidase (Vector Laboratories, Burlingame, CA) was applied to react with the secondary antibody. The substrate 3-amino-9-ethyl carbazole (Vector Laboratories) was then added to develop peroxidase activity as a red color. The slide was counterstained with hematoxylin and mounted for examination under a microscope (Zeiss).

### Animal study to examine the role of CCL20 in parturition

C57BL/6 mice (Charles River, Beijing, China) were used following accepted standards for animal care, which was approved by the Institutional Review Board of Ren Ji Hospital, School of Medicine, Shanghai Jiao Tong University. Mice aging from 10 to 13 weeks were mated. When a vaginal plug was present, it was counted as 0.5 days post-coitus (dpc). To observe whether injection of CCL20 could induce preterm birth, recombinant CCL20 (10 μg/kg BW) or equivalent maximal amount of LPS (1 ng/kg BW) remained in the preparation of recombinant CCL20 was injected intraperitoneally on 17 dpc. Some of the mice were allowed to deliver spontaneously for observation of delivery time and fetal demise, and some were sacrificed 12 h after injection for collection of fetal membranes, uterus, decidua and placentae to examine the infiltration of leukocytes with immunohistochemical staining of CD45 as described above with a CD45 antibody (Cell signaling; 70257S) at 1:100 dilution.

### Statistical analysis

Paired or unpaired Student’s t-tests where appropriate was used to assess the difference between TL_P and TL_D groups or between TL_P and TNL_P groups in terms of subcluster proportion and CCL20 expression. Unpaired Student’s t-test was performed to compare the delivery time of pregnant mice with or without CCL20 injection. Quantitative data are presented as mean ± SEM. Significance was set at P < 0.05.

## Supplementary Information

**Additional file 1: Fig. S1**. Information of individual human amnion sample. **Fig. S2**. Expression of established markers in six cell types of the human amnion. **Fig. S3**. Feature plots of immune cell marker expression in immunocytes of the human amnion. **Fig. S4**. GO analysis of highly-expressed genes in subclusters 1 and 4 of immunocytes of the human amnion. **Fig. S5**. Top DEGs in individual subclusters of EpC_FB of the human amnion. **Fig. S6**. Immunohistochemical staining of CCL20 in the human amnion in TNL_P, TL_D and TL_P groups. **Fig. S7**. Negative control of immunohistochemical staining of CD45 in mouse intrauterine tissues. **Table S1**. Information of each individual sample. **Table S2**. Cell number and proportion of each cell type. **Table S3**. Demographic and clinical characteristics of recruited pregnant women.

**Additional file 2: Dataset 1**. DEGs in epithelial cells between TL_P and TNL_P groups. **Dataset 2**. DEGs in epithelial cells between TL_P and TL_D groups. **Dataset 3**. DEGs in fibroblast cells between TL_P and TNL_P groups. **Dataset 4**. DEGs in fibroblast cells between TL_P and TL_D groups. **Dataset 5**. DEGs in immunocytes between TL_P and TNL_P groups. **Dataset 6**. DEGs in immunocytes between TL_P and TL_D groups. **Dataset 7**. Intersection of DEGs between TL_P and TNL_P groups with DEGs between TL_P and TL_D groups in epithelial cells. **Dataset 8**. Intersection of DEGs between TL_P and TNL_P groups with DEGs between TL_P and TL_D groups in fibroblast cells. **Dataset 9**. Intersection of DEGs between TL_P and TNL_P groups with DEGs between TL_P and TL_D groups in immunocytes. **Dataset 10**. Specific DEGs in epithelial cells between TL_P and TL_D groups, TL_P and TNL_P groups. **Dataset 11**. Specific DEGs in fibroblast cells between TL_P and TL_D groups, TL_P and TNL_P groups. **Dataset 12**. Specific DEGs in immunocytes between TL_P and TL_D groups, TL_P and TNL_P groups.

## Data Availability

The data used to support the findings of this study are available from the corresponding author upon reasonable request.

## Abbreviations

ZAM: The zone of altered morphology
scRNA-seq: Single cell RNA sequencing
EMT: Epithelial-mesenchymal transition
EIT: Epithelial-immune transition
MIT: Mesenchymal-immune transition
CCL20: C–C motif chemokine ligand 20
TL: Term labor
TNL: Term non-labor
TL_P: Term labor-proximal
TL_D: Term labor-distal
TNL_P: Term non-labor-proximal
EpC: Epithelial cells
FB: Fibroblasts
ImC: Immunocytes
EpC_FB: Epithelial_fibroblast cells
ImC_EpC: Immune_epithelial cells
ImC_FB: Immune_fibroblasts
DEGs: Differentially expressed genes
GO: Gene ontology
TFs: Transcriptional factors
PBS: Phosphate-buffered saline
GEM: Genetically engineered model
UMI: Unique molecular identifier
MNN: Mutual nearest neighbors
PCA: Principal component analysis
tSNE: T-distributed stochastic neighbor embedding
SCENIC: Single-cell regulatory network inference and clustering
qRT-PCR: Quantitative real-time PCR
ELISA: Enzyme-Linked immunosorbent assay
KLK: Kallikrein
MMPs: Matrix metalloproteinases
MGST1: Microsomal glutathione S-transferase 1
CCR6: CC chemokine receptor 6
BW: Body weight
AQP9: Aquaporin 9
