# Supplementary material for: Single cell transcriptomic analysis of human amnion identifies cell-specific signatures associated with membrane rupture and parturition
Source: Cell Biosci. 2022 May 18;12:64. doi: 10.1186/s13578-022-00797-4 (PMC9118831; doi:10.1186/s13578-022-00797-4)
Supplement: Supplementary file 1 — Additional file 1: Fig. S1. Information of individual human amnion sample. Fig. S2. Expression of established markers in six cell types of the human amnion. Fig. S3. Feature plots of immune cell marker expression in immunocytes of the human amnion. Fig. S4. GO analysis of highly-expressed genes in subclusters 1 and 4 of immunocytes of the human amnion. Fig. S5. Top DEGs in individual subclusters of EpC_FB of the human amnion. Fig. S6. Immunohistochemical staining of CCL20 in the human amnion in TNL_P, TL_D and TL_P groups. Fig. S7. Negative control of immunohistochemical staining of CD45 in mouse intrauterine tissues. Table S1. Information of each individual sample. Table S2. Cell number and proportion of each cell type. Table S3. Demographic and clinical characteristics of recruited pregnant women. [file 13578_2022_797_MOESM1_ESM.docx]

Supplementary Materials for

**Single cell transcriptomic analysis of human amnion identifies cell-specific signatures associated with membrane rupture and parturition**

Wang-Sheng Wang^1,2*^, Yi-Kai Lin^1,2^, Fan Zhang^1,2^, Wen-Jia Lei^1,2^, Fang Pan^1,2^, Ya-nan Zhu^1,2^, Jiang-wen Lu^1,2^, Chu-Yue Zhang^1,2^, Qiong Zhou^3^, Hao Ying^4^, Kang Sun^1,2*^

*Corresponding author. Dr. Kang Sun, [sungangrenji@hotmail.com](mailto:sungangrenji@hotmail.com) or Dr. Wang-sheng Wang, [wangsheng_wang@hotmail.com](mailto:wangsheng_wang@hotmail.com),

**Contents:**

**Fig. S1** Information of individual human amnion sample.

**Fig. S2** Expression of established markers in six cell types of the human amnion.

**Fig. S3** Feature plots of immune cell marker expression in immunocytes of the human amnion.

**Fig. S4** GO analysis of highly-expressed genes in subclusters 1 and 4 of immunocytes of the human amnion.

**Fig. S5** Top DEGs in individual subclusters of EpC_FB of the human amnion.

**Fig. S6** Immunohistochemical staining of CCL20 in the human amnion in TNL_P, TL_D and TL_P groups.

**Fig. S7** Negative control of immunohistochemical staining of CD45 in mouse intrauterine tissues.

**Table S1** Information of each individual sample.

**Table S2** Cell number and proportion of each cell type.

**Table S3** Demographic and clinical characteristics of recruited pregnant women.

**Dataset 1** DEGs in epithelial cells between TL_P and TNL_P groups.

**Dataset 2** DEGs in epithelial cells between TL_P and TL_D groups.

**Dataset 3** DEGs in fibroblast cells between TL_P and TNL_P groups.

**Dataset 4** DEGs in fibroblast cells between TL_P and TL_D groups.

**Dataset 5** DEGs in immunocytes between TL_P and TNL_P groups.

**Dataset 6** DEGs in immunocytes between TL_P and TL_D groups.

**Dataset 7** Intersection of DEGs between TL_P and TNL_P groups with DEGs between TL_P and TL_D groups in epithelial cells.

**Dataset 8** Intersection of DEGs between TL_P and TNL_P groups with DEGs between TL_P and TL_D groups in fibroblast cells.

**Dataset 9** Intersection of DEGs between TL_P and TNL_P groups with DEGs between TL_P and TL_D groups in immunocytes.

**Dataset 10** Specific DEGs in epithelial cells between TL_P and TL_D groups, TL_P and TNL_P groups.

**Dataset 11** Specific DEGs in fibroblast cells between TL_P and TL_D groups, TL_P and TNL_P groups.

**Dataset 12** Specific DEGs in immunocytes between TL_P and TL_D groups, TL_P and TNL_P groups.


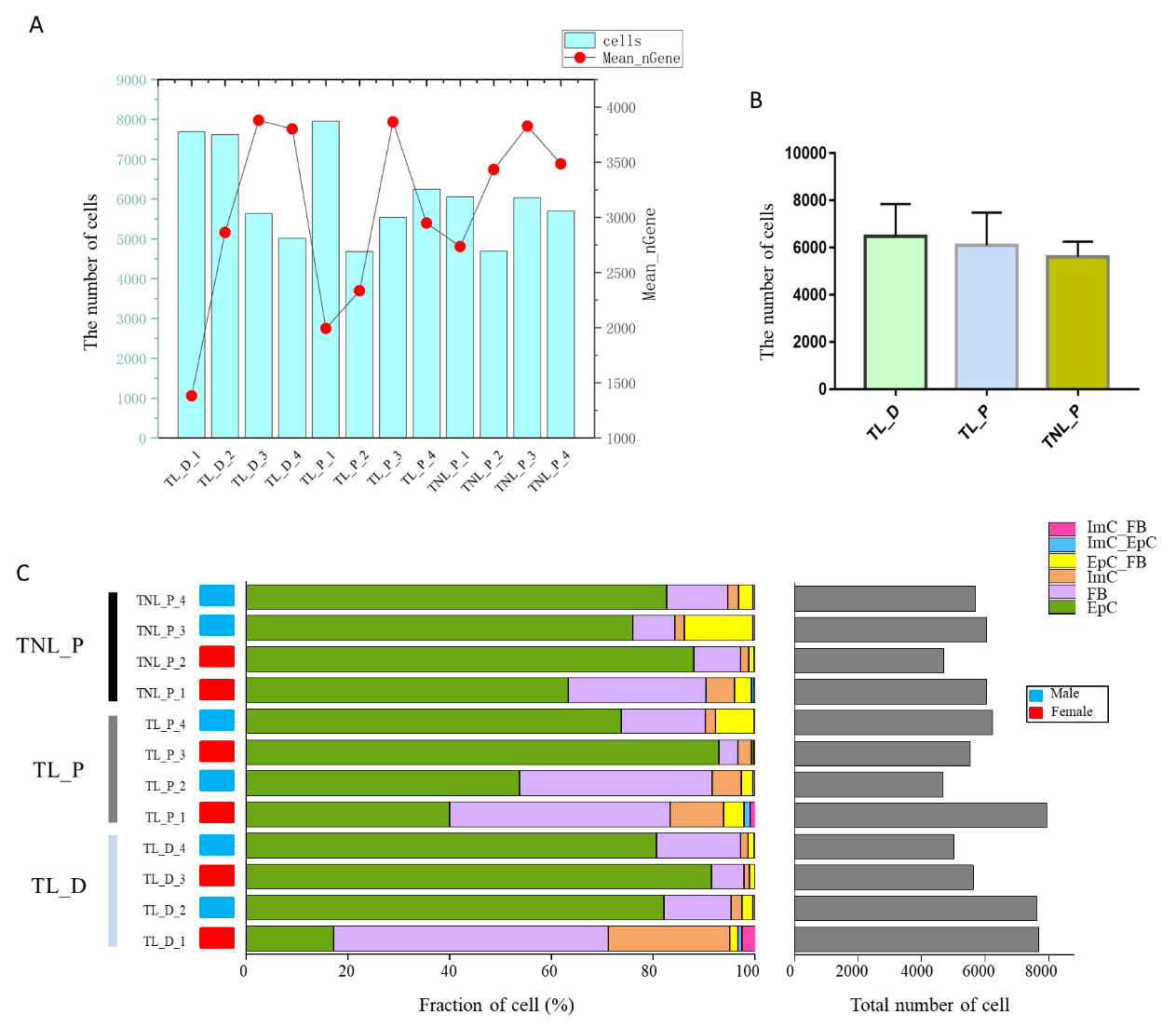


**Fig. S1 Information of individual human amnion sample.** **(A)** The cell number and the mean of gene number (mean_nGene) of individual samples. **(B)** The average number of cells of TL_D, TL_P and TNL_P groups. **(C)** Fractions of cell types of individual samples. TNL_P, term non-labor-proximal; TL_P, term labor-proximal (ZAM zone); TL_D, term labor-distal (non-ZAM zone). FB, fibroblasts; EpC, epithelial cells; ImC, immunocytes; EpC_FB, epithelial_fibroblasts; ImC_EpC, immune_epithelial cells and ImC_FB, immune_fibroblasts.


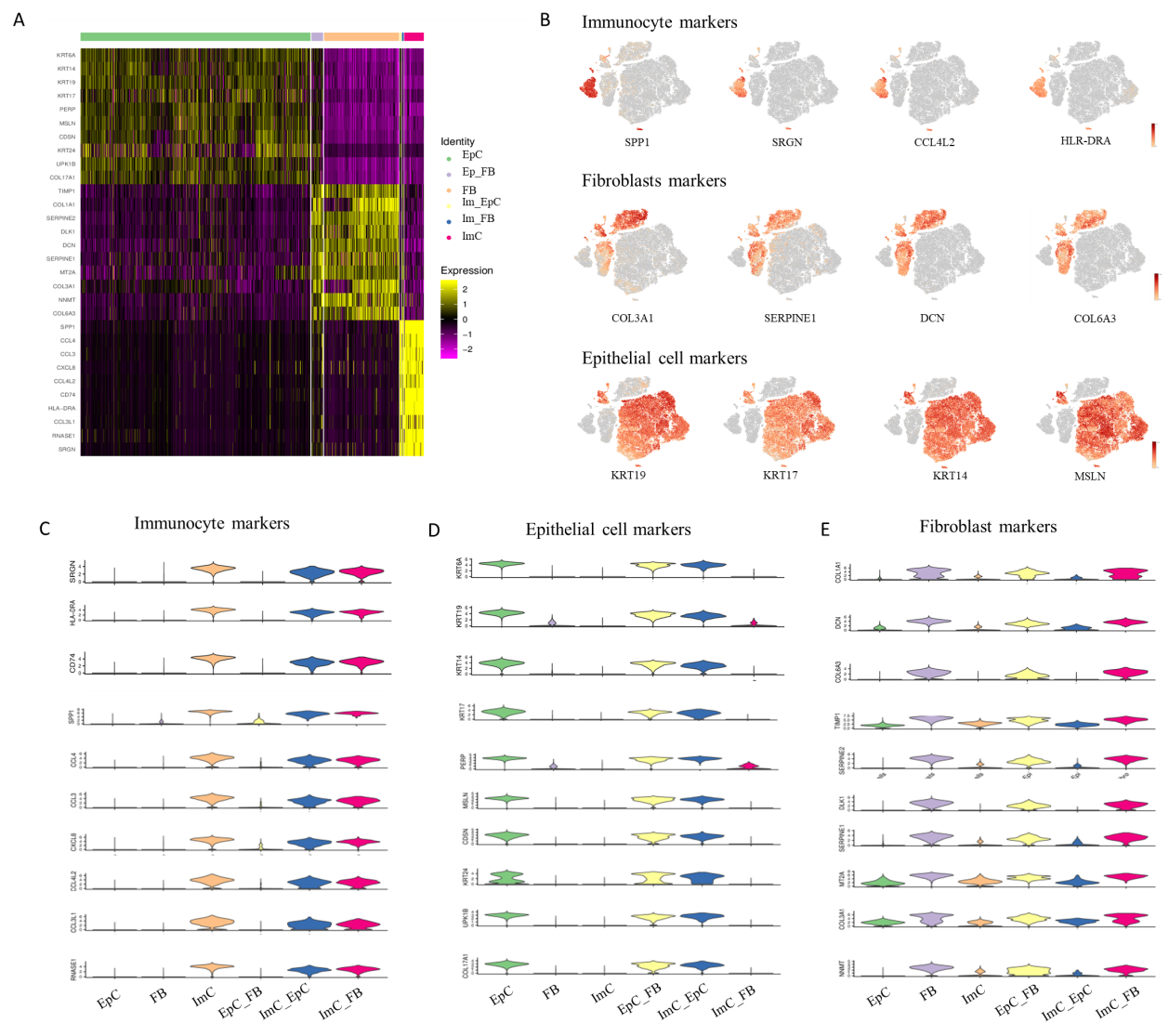


**Fig. S2 Expression of established markers in six cell types of the human amnion. H**eatmap (A), feature plots (B) and violin plots (C-E) show the expression of established markers of epithelial cells, fibroblasts and immunocytes. FB, fibroblasts; EpC, epithelial cells; ImC, immunocytes; EpC_FB, epithelial_fibroblasts; ImC_EpC, immune_epithelial cells and ImC_FB, immune_fibroblasts.


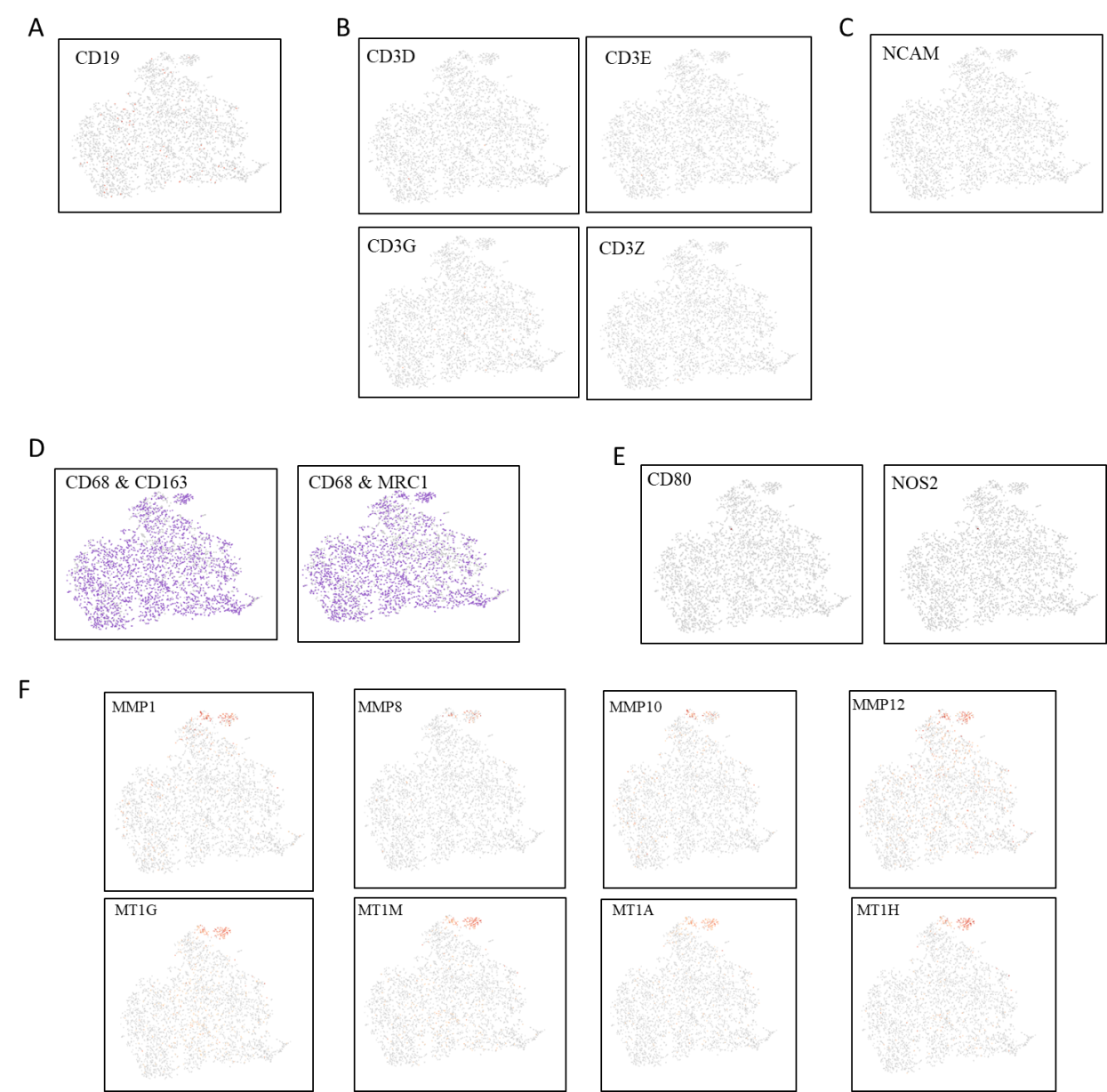


**Fig. S3 Feature plots of immune cell marker expression in immunocytes of the human amnion.** **(A-C)** Feature plots show the expression of markers of B cell (CD19) (A), T cell (CD3) (B) and NK cell (NCAM) (C). **(D)** Feature plots display co-expression of M1 (CD68) and M2 (CD163, MRC1) subtypes of macrophages. **(E)** Feature plots show the expression of M1 markers (CD80, NOS2). **(F)** Feature plots show the expression of matrix metalloproteinases (MMPs) and metallothioneins (MTs) in subcluster 6.


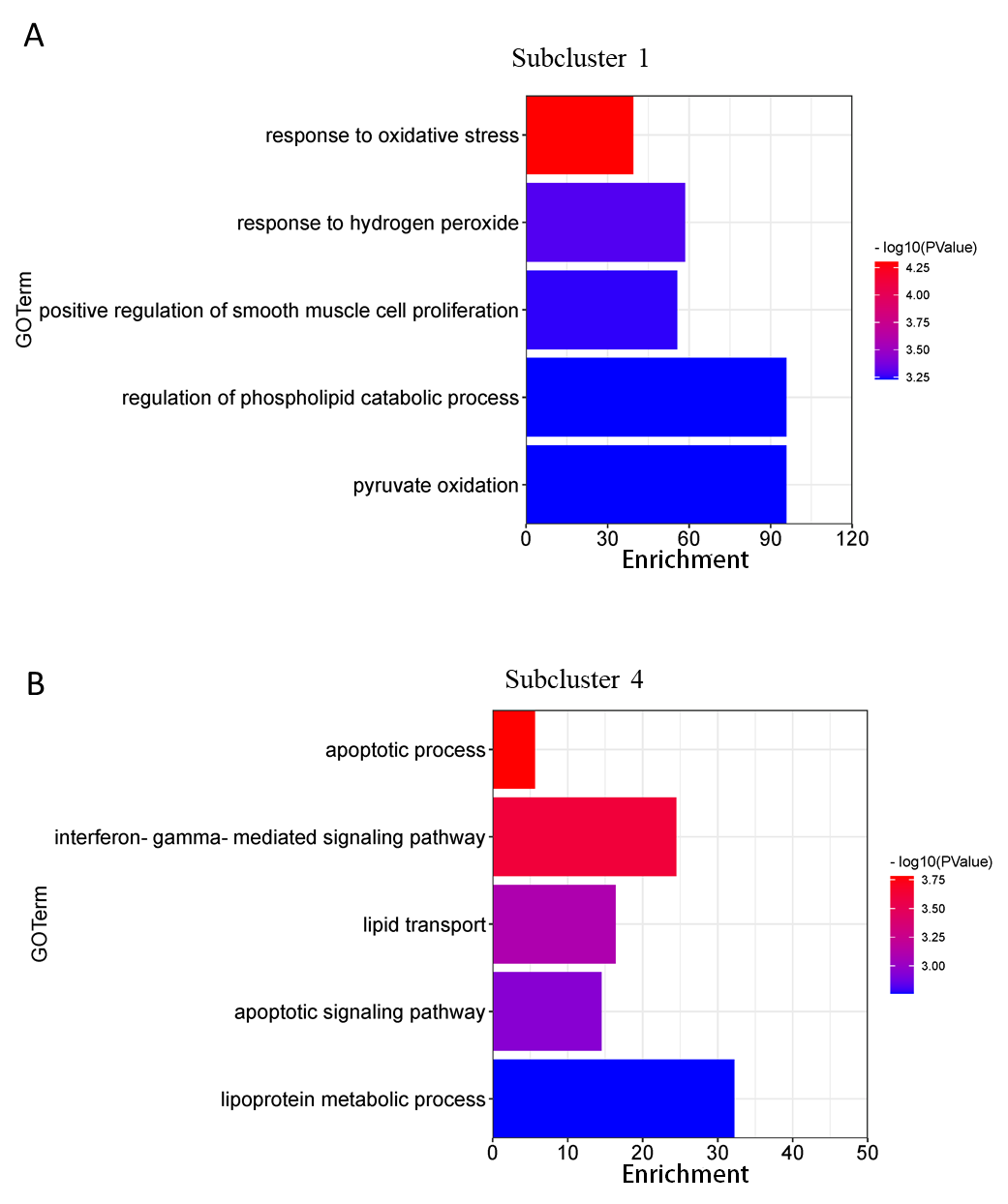


**Fig. S4 GO analysis of highly-expressed genes in subclusters 1 and 4 of immunocytes of the human amnion.** The top highly-expressed genes in subclusters 1 (A) and 4 (B) were related to oxidative stress and apoptotic process respectively.


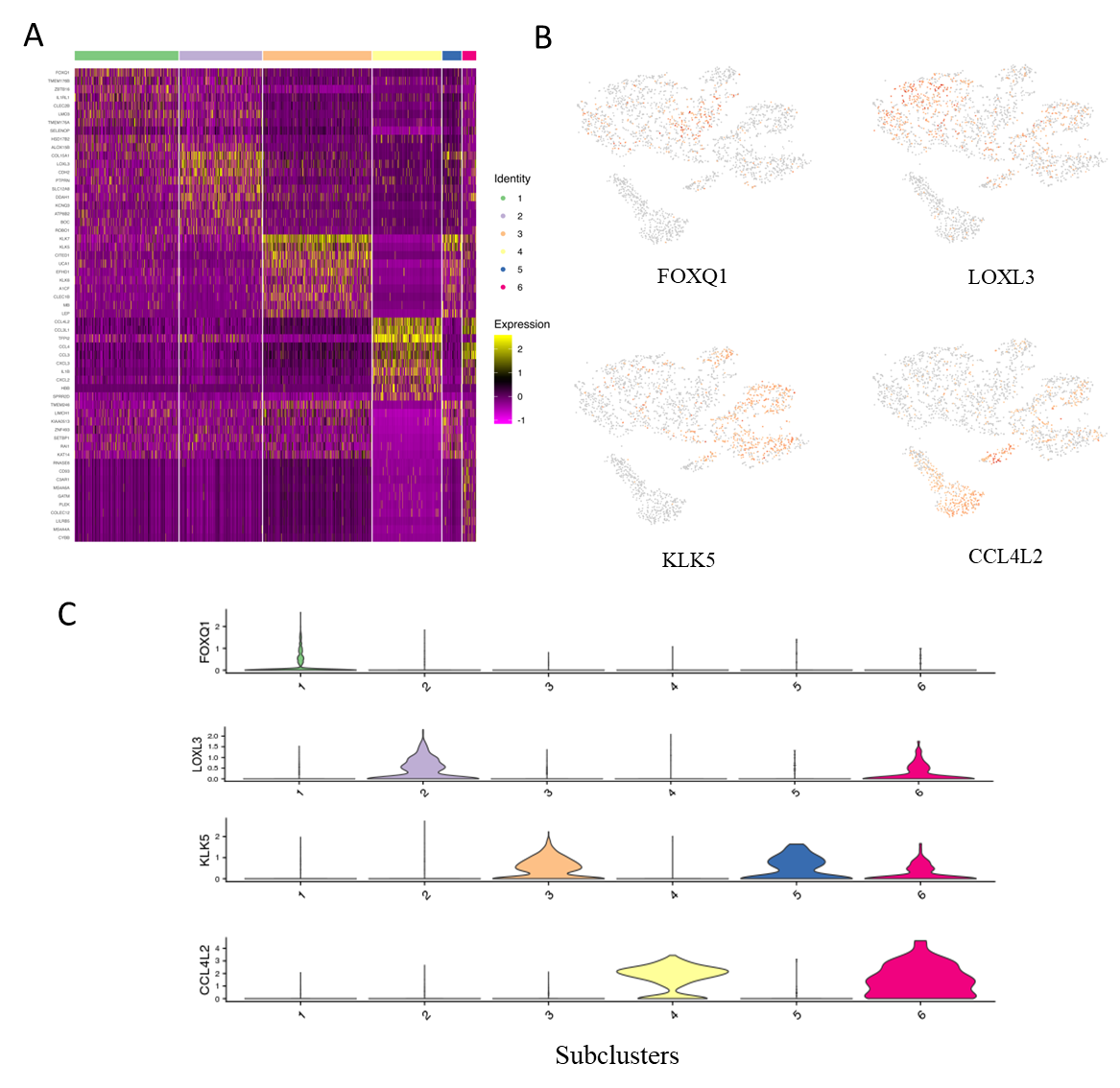


**Fig. S5 Top DEGs in individual subclusters of EpC_FB of the human amnion.** **(A)** Heatmap displays top 10 DEGs in each individual subcluster. **(B and C)** Feature plots (B) and violin plots (C) show the expression of FOXQ1, LOXL3, KLK5 and CCL4L2 in individual subclusters.


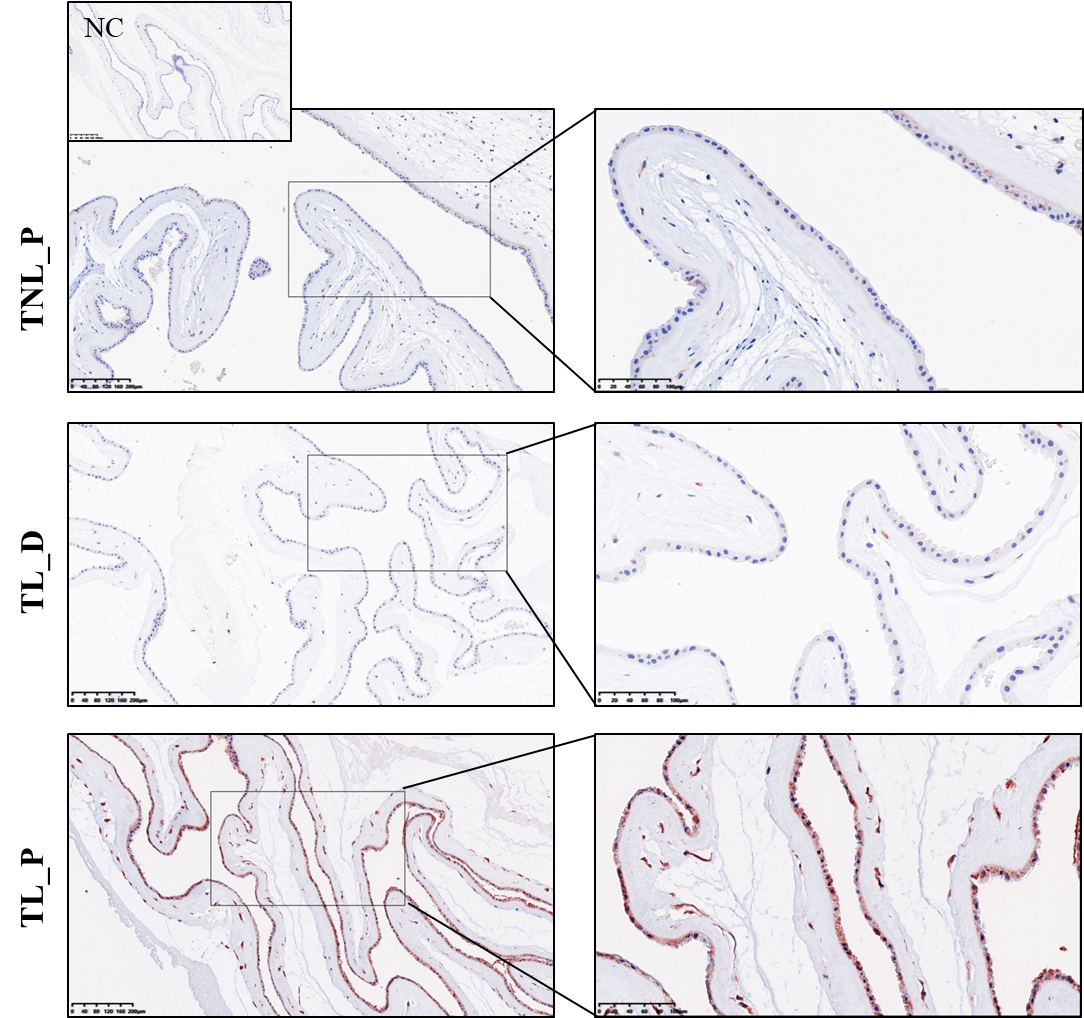


**Fig. S6 Representative images of immunohistochemical staining of CCL20 in human amnion in TNL_P, TL_D and TL_P groups.** Increased CCL20 abundance was observed in TL_P group. NC, negative control. TNL_P, term non-labor-proximal; TL_P, term labor-proximal (ZAM zone); TL_D, term labor-distal (non-ZAM zone). n=3.


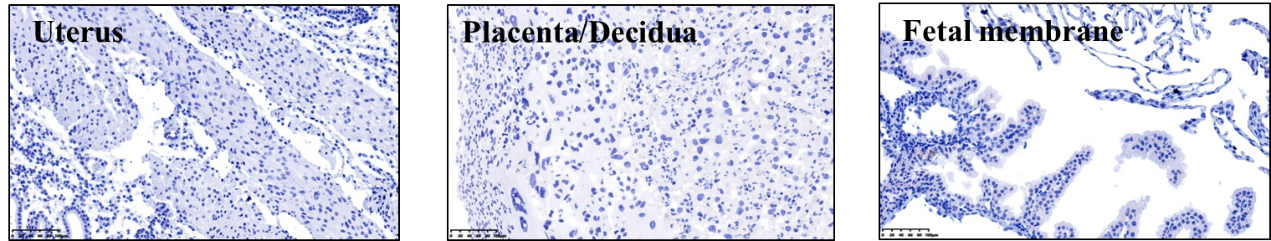


**Fig. S7 Negative control of immunohistochemical staining of CD45 in mouse intrauterine tissues.**

**Table S1 Information of each individual sample**

| **Sample ID** | **Total cell** | **Mean UMI** | **Location** | **Condition** | **Fetal gender** |
| --- | --- | --- | --- | --- | --- |
| TL_D_1 | 7686 | 3704 | Non-ZAM | TL | Female |
| TL_D_2 | 7612 | 8012 | Non-ZAM | TL | Male |
| TL_D_3 | 5628 | 15723 | Non-ZAM | TL | Female |
| TL_D_4 | 5014 | 20165 | Non-ZAM | TL | Male |
| TL_P_1 | 7952 | 6232 | ZAM | TL | Female |
| TL_P_2 | 4679 | 7238 | ZAM | TL | Male |
| TL_P_3 | 5536 | 16645 | ZAM | TL | Female |
| TL_P_4 | 6243 | 14111 | ZAM | TL | Male |
| TNL_P_1 | 6052 | 12927 | ZAM | TNL | Female |
| TNL_P_2 | 4846 | 13449 | ZAM | TNL | Female |
| TNL_P_3 | 6032 | 15975 | ZAM | TNL | Male |
| TNL_P_4 | 5698 | 18496 | ZAM | TNL | Male |

**Table S2 Cell number and proportion of each cell type**

|  | **TNL_P** | **TL_P** | **TL_D** | **Total** |
| --- | --- | --- | --- | --- |
| EpC | 17240 (77.45) | 15444 (65.10) | 16765 (67.85) | 49449 (67.91) |
| FB | 3262 (14.18) | 6452 (25.35) | 6337 (22.51) | 16051 (22.04) |
| ImC | 640 (2.77) | 1378 (5.23) | 2122 (7.11) | 4140 (5.69) |
| EpC_FB | 1224 (5.17) | 917 (3.55) | 403 (1.49) | 2544 (3.49) |
| ImC_EpC | 88 (0.38) | 128 (0.46) | 108 (0.37) | 324 (0.45) |
| ImC_FB | 14 (0.06) | 91 (0.30) | 205 (0.67) | 310 (0.43) |

Number in brackets indicates percentage of this cell type. TNL_P, term non labor-proximal; TL_P, term labor-proximal (ZAM zone); TL_D, term labor-distal (non-ZAM zone).

**Table S3 Demographic and clinical characteristics of recruited pregnant women**

| **Demographic features** | **TNL (n=18)** | **TL (n=18)** | ***P* value** |
| --- | --- | --- | --- |
| Maternal age (year) | 32.10+2.56 | 29.25+4.5 | 0.30 |
| Gestational age at delivery (week) | 38.71+0.39 | 38.95+0.61 | 0.17 |
| Labor status |  |  |  |
| Spontaneous (%) | 0 | 100% | N/A |
| Induced (%) | 0 | 0 | N/A |
| Delivery mode |  |  |  |
| Vaginal (%) | 0 | 100% | N/A |
| C-section (%) | 100% | 0 | N/A |
| Membrane rupture |  |  |  |
| SPOM (%) | 0 | 100% | N/A |
| ARM (%) | 100% | 0 | N/A |
| Gravidity median (range) | 2 (1-4) | 2 (1-3) | 0.55 |
| Parity median (range) | 2 (1-2) | 1 (1-2) | 0.03 |
| Fetal gender (male/female) | 7/11 | 8/10 | 0.91 |
| Birth weight (g) | 3414.44+343.15 | 3295.84+275.78 | 0.26 |
| Length of labor (hour) | N/A | 6.67+2.68 | N/A |

ARM, artificial rupture of membranes; C-section, caesarean section; SROM, spontaneous rupture of membranes with labor.
